# Isolation of *amaranthin synthetase* from *Chenopodium quinoa* and construction of an amaranthin production system using suspension‐cultured tobacco BY‐2 cells

**DOI:** 10.1111/pbi.13032

**Published:** 2018-12-05

**Authors:** Tomohiro Imamura, Noriyoshi Isozumi, Yasuki Higashimura, Akio Miyazato, Hiroharu Mizukoshi, Shinya Ohki, Masashi Mori

**Affiliations:** ^1^ Research Institute for Bioresources and Biotechnology Ishikawa Prefectural University Nonoichi Ishikawa Japan; ^2^ Center for Nano Materials and Technology (CNMT) Japan Advanced Institute of Science and Technology (JAIST) Nomi Ishikawa Japan; ^3^ Department of Food Science Ishikawa Prefectural University Nonoichi Ishikawa Japan; ^4^ Technology Development Group, Actree Co. Hakusan Ishikawa Japan

**Keywords:** *Amaranthin synthetase*, betalain, HIV‐1 protease inhibitor, MCF‐7 cells, quinoa, tobacco BY‐2 cells

## Abstract

Betalains are plant pigments primarily produced by plants of the order Caryophyllales. Because betalain possesses anti‐inflammatory and anticancer activities, it may be useful as a pharmaceutical agent and dietary supplement. Recent studies have identified the genes involved in the betalain biosynthesis of betanin. Amaranthin and celosianin II are abundant in the quinoa (*Chenopodium quinoa* Willd.) hypocotyl, and amaranthin comprises glucuronic acid bound to betanin; therefore, this suggests the existence of a glucuronyltransferase involved in the synthesis of amaranthin in the quinoa hypocotyl. To identify the gene involved in amaranthin biosynthesis, we performed a BLAST analysis and phylogenetic tree analysis based on sequences homologous to flavonoid glycosyltransferase, followed by expression analysis on the quinoa hypocotyl to obtain three candidate proteins. Production of amaranthin in a transient *Nicotiana benthamiana* expression system was evaluated for these candidates and one was identified as having the ability to produce amaranthin. The gene encoding this protein was *quinoa amaranthin synthetase 1* (*CqAmaSy1*). We also created a transgenic tobacco bright yellow‐2 (BY‐2) cell line wherein four betalain biosynthesis genes were introduced to facilitate amaranthin production. This transgenic cell line produced 13.67 ± 4.13 μm (mean ± SEM) amaranthin and 26.60 ± 1.53 μm betanin, whereas the production of isoamaranthin and isobetanin could not be detected. Tests confirmed the ability of amaranthin and betanin to slightly suppress cancer cell viability. Furthermore, amaranthin was shown to significantly inhibit HIV‐1 protease activity, whereas betanin did not.

## Introduction

Betalains are tyrosine‐derived red–violet and yellow pigments found exclusively in plants of the order Caryophyllales, and crops such as beets (*Beta vulgaris*), quinoa (*Chenopodium quinoa* Willd.) and amaranth (*Amaranthus hypochondriacus*) contain these pigments. Unlike flavonoids and carotenoids, betalains contain nitrogen, and they cannot naturally coexist with anthocyanins in the same plant (Stafford, 1994). They are divided into two groups, betacyanins (red and purple) and betaxanthins (yellow and orange), and are found in various plant tissues, such as leaves, stems, roots and flowers. Previous studies have demonstrated that betalains exhibit strong antioxidant activity (Wybraniec *et al*., 2011) and are involved in responses to both stress and environmental stimuli in plants (Jain *et al*., 2015; Polturak *et al*., 2017). In addition, it has been suggested that they play a role in attracting pollinators to flowers (Gandia‐Herrero *et al*., 2005).

**Figure 1:**
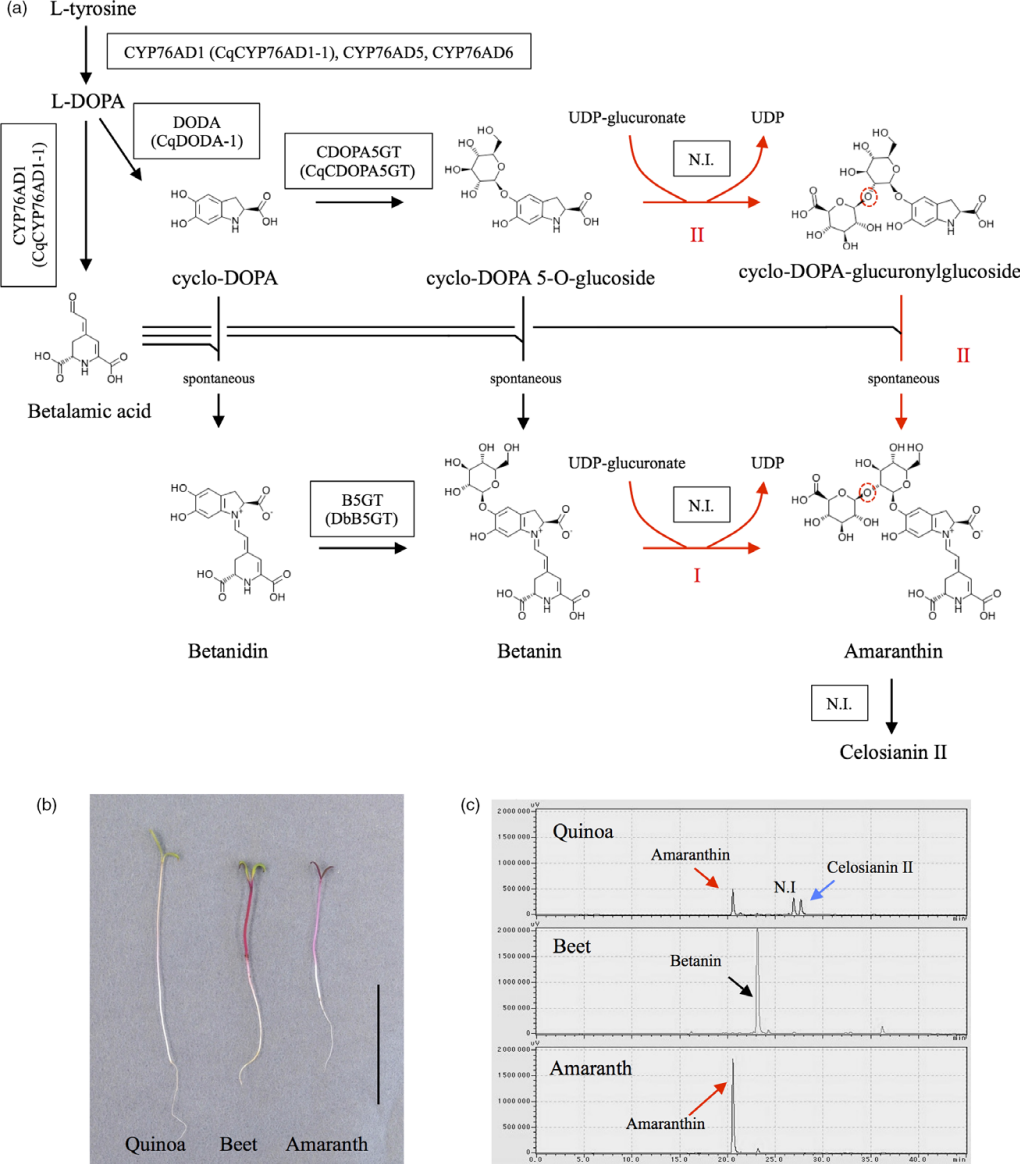
Pigment analysis of quinoa and beet hypocotyls. (a) Scheme for the betacyanin biosynthetic pathway. Boxes indicate the betalain biosynthetic enzyme. Red arrows indicate amaranthin biosynthetic pathways (I, II). CYP76AD1, cytochrome P450 76AD1; DODA, DOPA 4,5‐dioxygenase; CDOPA5GT, cyclo‐DOPA 5‐*O*‐glucosyltransferase; B5GT, betanidin 5‐*O*‐glucosyltransferase; N.I., not identified. Cq, *Chenopodium quinoa*; Db, *Dorotheanthus bellidiformis*. Enzymes in parenthesis were used in this study. Red‐dashed circles indicate β‐1,2‐glycosidic bond. (b) Photographs of 5‐day‐old quinoa, beet and amaranth seedlings. Bar = 4 cm. (c) Production of betalain pigments in quinoa, beet and amaranth hypocotyls. The upper, middle and lower panels show the chromatogram of the quinoa, beet and amaranth of hypocotyl extracts respectively. These chromatograms monitored at 536 nm. Red, black and blue arrows indicate amaranthin, betanin and celosianin II respectively. N.I. indicates nonidentified peak. The horizontal axis indicates the retention time (min), whereas the vertical axis indicates the signal intensity (μV).

Recent studies have elucidated the betalain biosynthetic pathway (Figure 1a), which begins with the hydroxylation of L‐tyrosine to form L‐3,4‐dihydroxyphenylalanine (l‐DOPA) by the redundant cytochrome P450 enzyme CYP76AD1, CYP76AD5 and CYP76AD6 (Polturak *et al*., 2016; Sunnadeniya *et al*., 2016). L‐DOPA is then converted to betalamic acid by DOPA 4,5‐dioxygenase (Christinet *et al*., 2004; Gandia‐Herrero and Garcia‐Carmona, 2012) or to cyclo‐DOPA by CYP76AD1 (Hatlestad *et al*., 2012). Betalamic acid can further spontaneously condense with amino acids or other amine‐containing compounds to form yellow fluorescent betaxanthin pigments (Schliemann *et al*., 1999) or with cyclo‐DOPA to form red betacyanin pigments (Steiner *et al*., 1999). Betacyanins can be further modified by betalain‐related glucosyltransferases that catalyse the 5‐*O*‐glucosylation of cyclo‐DOPA (Sasaki *et al*., 2005) or alternatively the 5‐*O*‐ or 6‐*O*‐glucosylation of betanidin (Figure 1a, Das *et al*., 2013; Vogt, 2002; Vogt *et al*., 1999). Recently, our group and others sequenced the quinoa genome (Jarvis *et al*., 2017; Yasui *et al*., 2016; Zou *et al*., 2017), and based on these genomic data, we identified the genes responsible for betalain biosynthesis including *CqCYP76AD1*,* CqDODA* and *CqCDOPA5GT* (Imamura *et al*., 2018).

Betalains are often extracted from plant and used as food additives because of their vivid colour. In particular, beetroot extract, which is designated by the E162 label, is used by the food industry and approved by the U.S. Food and Drug Administration (USFDA) and European Union regulatory agencies as a natural colorant. Because this colorant is a natural product derived from food crops, its toxicity to humans is extremely low. Betalain‐rich beetroot concentrates used as dietary supplements have been shown to improve exercise performance and recovery in competitive triathletes (Montenegro *et al*., 2017).

Basic medical research has revealed that betalain‐rich extracts possess anti‐inflammatory and anticancer properties (Kapadia *et al*., 1996; Lechner *et al*., 2010; Martinez *et al*., 2015; Rodriguez *et al*., 2016). Although more than 50 molecular species of betalains have been reported (Delgado‐Vargas *et al*., 2000), very few exhibit biological activity. However, betanin (betanidin 5‐*O*‐β‐glucoside), a major red pigment of beetroot extract, has been demonstrated to inhibit low‐density lipoprotein oxidation (Tesoriere *et al*., 2003, 2004) and to induce apoptosis and autophagic cell death in human cancer cells (Nowacki *et al*., 2015). Similarly, indicaxanthin, a yellow pigment produced by the condensation of proline and betalamic acid, has been reported to possess anti‐inflammatory properties (Allegra *et al*., 2014) and to be antiproliferative and pro‐apoptotic in human cancer cells (Naselli *et al*., 2014). Based on these findings, betalain pigments hold promise as potential pharmaceuticals.

The biological mechanisms of the action of many betalain pigments remain unknown; therefore, evaluation of the pharmaceutical potential of individual betalain pigments requires the preparation of sufficient amounts of each individual substance for subsequent analysis. Historically, the preparation of a single pure betalain pigment requires extraction and purification from betalain‐producing plants. This was problematic as plant procurement was not always easy and substantial time and effort were required to purify the substance of interest. In recent years, and as an alternative to using betalain‐producing plants, artificial betalain pigments have been developed by introducing betalain biosynthetic genes into non‐betalain‐producing plants or cultured plant cells, such as tobacco bright yellow‐2 (BY‐2) cells (Polturak *et al*., 2017). Because BY‐2 cells can be easily cultured both homogeneously and aseptically, they can be used for mass production and are suitable for use in the production of biopharmaceutical products (Doran, 2000; Hellwig *et al*., 2004). In addition, our laboratory has succeeded in using these cells to produce betanin using betalain biosynthetic genes from quinoa (Imamura *et al*., 2018). However, because most betalain‐modification enzymes have not been identified, only a few betalain pigments such as betanin and isobetanin, can be produced using this artificial culture system.

Amaranthin, a betanidin 5‐*O*‐β‐glucuronosylglucoside (Figure 1a), is a major betalain pigment that accumulates in the quinoa hypocotyl (Figure 1b,c; Imamura *et al*., 2018). Its biosynthesis is predicted to occur through two pathways; via direct conjugation between glucuronic acid and betanin (pathway I) or via conjugation between glucuronic acid and *cyclo*‐DOPA 5‐*O*‐glucoside followed by condensation with betalamic acid (pathway II; Figures 1a and S1). While it has been reported that cyclo‐DOPA 5‐*O*‐glucoside is a more effective substrate than betanidin in amaranthin synthesis (Sciuto *et al*., 1974), amaranthin synthetase (AmaSy) has not yet been isolated. Therefore, amaranthin might possess biological effects that have not previously been reported.

To identify new biological activities of individual betalain pigments, we isolated amaranthin synthetase from quinoa and constructed a betalain production system using BY‐2 cells. In this study, we used phylogenetic analysis to search for genes involved in amaranthin synthesis and succeeded in isolating two genes that encode amaranthin synthetase. By introducing these betalain biosynthetic genes, we succeeded for the first time in producing amaranthin in BY‐2 cells. In addition, we used this amaranthin to identify new biological activities of the molecule. Thus, our isolation of the amaranthin synthetase gene will both facilitate the discovery of new biological activities and lead to the development of artificial systems for industrial betacyanin production.

## Results

### Search for the amaranthin synthetase in the quinoa genome

**Figure 2:**
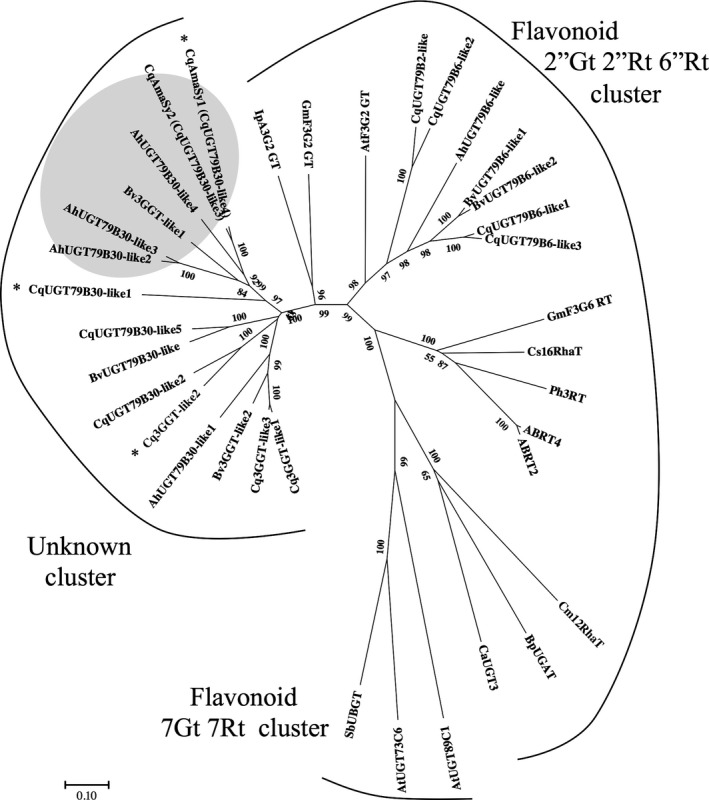
Molecular phylogenetic tree of some flavonoid glycosyltransferases based on amino acid sequences. Multiple sequences were aligned using ClustalW and were used for tree construction using the maximum likelihood method of MEGA7. Bootstrap values from 5000 replicates are shown on branches. The bar represents 0.1 amino acid substitutions per site. Asterisks indicate genes expressed in the quinoa hypocotyl. Grey shading denotes the amaranthin synthetase cluster. Details about the flavonoid glycosyltransferase homologs from other plant species are given in Table S1. Species abbreviations: Ah, *Amaranthus hypochondriacus*; At, *Arabidopsis thaliana*; Bp, *Bellis perennis*; Bv, *Beta vulgaris*; Ca, *Catharanthus roseus*; Cm, *Citrus maxima*; Cq, *Chenopodium quinoa*; Cs, *Citrus sinensis*; Gm, *Glycine max*; Ip, *Ipomoea purpurea*; Ph, *Petunia hybrida*; and Sb, *Scutellaria baicalensis*.

Amaranthin contains a β‐1,2‐glycosidic bond between its glucoside and glucuronic acid (Figures 1a and S1). Flavonoid‐glycoside glycosyltransferase, which is involved in the formation of β‐1,2‐glycosidic bonds between glucosides and glucose (Figure S1), has already been isolated from non‐betalain‐producing plants (Di *et al*., 2015; Morita *et al*., 2005; Yonekura‐Sakakibara *et al*., 2014); therefore, we attempted to identify the amaranthin synthetase based on homology with another flavonoid‐glycoside glycosyltransferase, namely *Arabidopsis* flavonoid 3‐*O*‐glucoside 2″‐*O*‐glucosyltransferase (UGT79B6, Accession No. NP_200212). Twelve genes exhibiting >40% protein homology with UGT79B6 and encoding full‐length proteins were identified in quinoa. Similarly, ten candidates were also identified in other betalain‐producing plants, such as beet and amaranth. We then performed a phylogenetic analysis on the selected proteins (Table S1). The phylogenetic tree separated into three clusters (flavonoid 2″Gt 2″Rt 6″Rt cluster, flavonoid 7Gt 7Rt cluster and unknown cluster). The flavonoid 2″Gt 2″Rt 6″Rt cluster represents a group of UDP‐glycosyltransferase that add sugars to the sugar moiety of flavonoid glycosides. The flavonoid 7Gt7Rt cluster is a group of UDP‐glycosyltransferases that catalyse glycosylation at the 7th position of the flavonoid aglycone. The unknown cluster remains an unknown UDP‐glycosyltransferase group. Four of the candidate quinoa proteins (CqUGT79B6‐like1, CqUGT79B6‐like2, CqUGT79B6‐like3 and CqUGT79B2‐like) were found to belong to the flavonoid 2″Gt 2″Rt 6″Rt cluster (Figure 2). The remaining eight candidates (Cq3GGT‐like1, Cq3GGT‐like2, Cq3GGT‐like3,CqUGT79B30‐like1, CqUGT79B30‐like2, CqUGT79B30‐like5, CqAmaSy1 and CqAmaSy2) belonged to an unknown cluster composed of proteins from betalain‐producing plants (Figure 2). There were four proteins from amaranth and three from beets in this cluster.

### Expression analysis of genes from the unknown cluster in the quinoa hypocotyl

**Figure 3:**
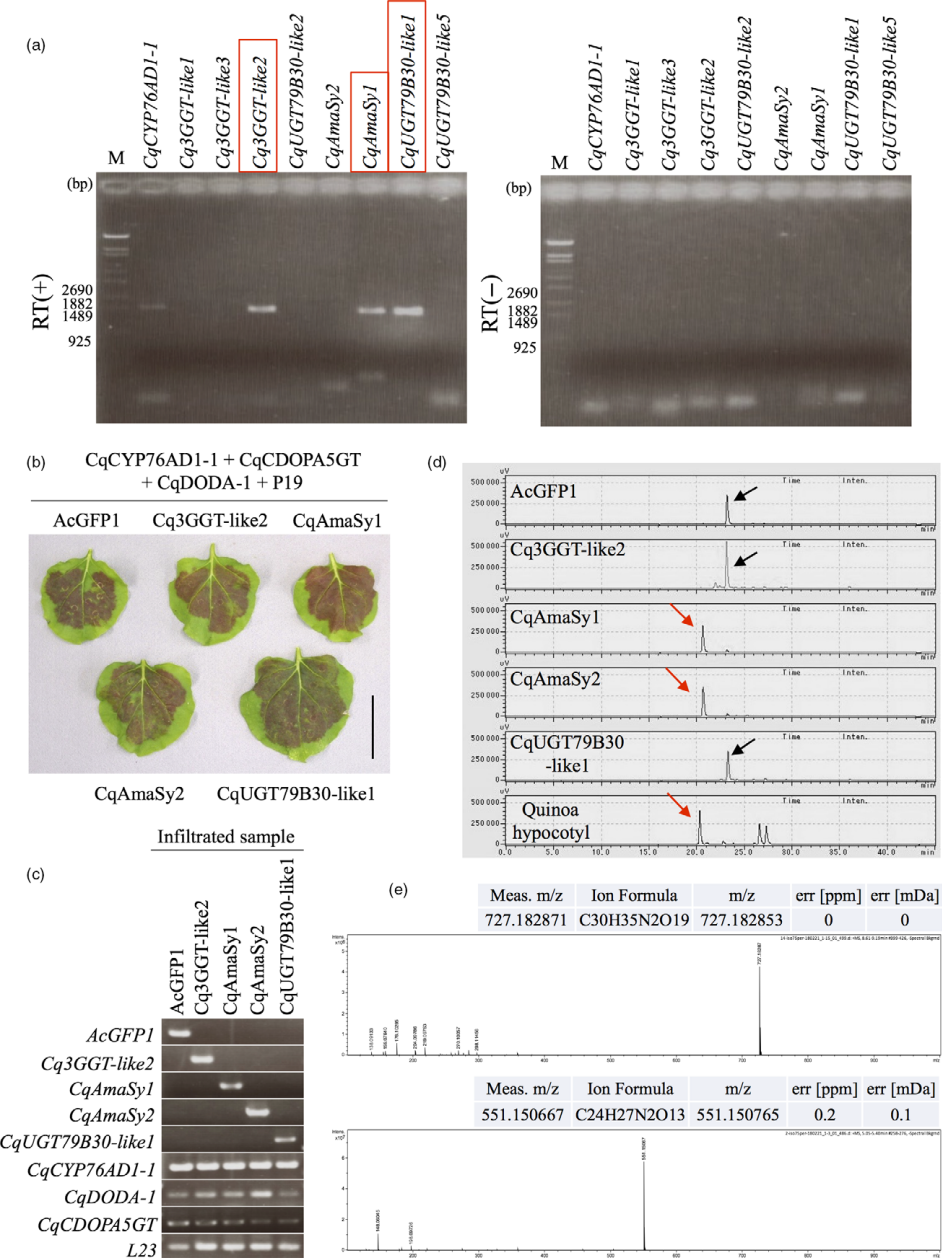
Identification of the amaranthin biosynthesis gene. (a) RT‐PCR analysis of candidate gene expression in the quinoa hypocotyl. RT (+) represents reverse‐transcribed samples (left panel), and RT (−) represents the corresponding reverse transcriptase‐free controls (right panel). Red boxes indicate expressed genes in quinoa hypocotyl. *CqCYP76AD1‐1* is used as a positive control. (b) Recombinant expression of candidate of amaranthin synthetase genes (*Cq3GGT‐like2*,* CqAmaSy1, CqAmaSy2* and *CqUGT79B30‐like1*) in *Nicotiana benthamiana* leaves. Co‐infiltration of transgenic *Agrobacterium* harbouring plasmids for the expression of the candidate gene with *CqCYP76AD1‐1, CqCDOPA5GT*,* CqDODA‐1* and P19. AcGFP1 is used as a negative control. Bar = 4 cm. (c) RT‐PCR analysis of the gene expression in infiltrated leaves of *N. benthamiana*. *L23* indicates an internal control. (d) HPLC chromatogram of the infected *N. benthamiana* leaf extract. Hypocotyl indicates the extract of the quinoa hypocotyl from CQ127 variety. Red and black arrows indicate amaranthin and betanin respectively. The horizontal axis indicates the retention time (min), whereas the vertical axis indicates the signal intensity (μV). (e) MS spectra of HPLC elution samples from *N. benthamiana* leaf extract. Upper and lower panels indicate HPLC elution samples of 21 (red arrows in c) and 24 (black arrows in c) min respectively. HPLC elution samples at 21 and 24 min indicate amaranthin and betanin respectively. The horizontal axis indicates mass‐to‐charge ratio (*m/z*). The vertical axis indicates the relative abundance.

Our group has recently reported that betalain pigments, putatively including amaranthin and celosianin II, accumulate in the quinoa hypocotyl (Figure 1b,c; Imamura *et al*., 2018). Furthermore, betalain biosynthesis genes, including *CqCYP76AD1‐1*,* CqDODA‐1* and *CqCDOPA5GT*, were expressed in quinoa hypocotyls (Imamura *et al*., 2018). These findings suggest that the gene encoding amaranthin synthetase is also expressed in quinoa hypocotyls. Therefore, eight genes belonging to the unknown cluster were examined for expression in quinoa hypocotyls. Consequently, three genes, including *Cq3GGT‐like2*,* CqUGT79B30‐like1* and *CqAmaSy1*, were found to be expressed in the form of the predicted full‐length open reading frame (ORF; Figure 3a). *CqAmaSy1a* and *CqAmaSy1b* have the same ORF and are adjacent to one another in the quinoa genome (Figure S2). Using *CqAmaSy1s* polymorphism in the 3′‐untranslated region (UTR) as an index, the nucleotide sequence of its reverse transcription product was examined, and only the *CqAmaSy1b* sequence was detected (Figure S2). These results suggest that the three candidate genes are expressed in the quinoa hypocotyl.

### Identification of amaranthin synthetase in quinoa

A system for the evaluation of betalain production by induced transient expression of candidate genes in the leaves of *Nicotiana benthamiana* was constructed in a previous study (Polturak *et al*., 2017). We recently succeeded in producing betanin, one of the precursors of amaranthin, by transiently expressing the genes for quinoa betalain biosynthesis (*CqCYP76AD1‐1*,* CqDODA‐1* and *CqCDOPA5GT*) in *N. benthamiana* leaves (Imamura *et al*., 2018); therefore, this system was used to evaluate the amaranthin synthetase activity of the three candidate genes.

An expression plasmid was constructed for each of the candidate genes (Figure S3) and introduced into *Agrobacterium*. Transformed *Agrobacterium* harbouring an overexpression vector for each of the candidate genes, *CqCYP76AD1‐1*,* CqDODA‐1* and *CqCDOPA5GT*, were co‐infiltrated into *N. benthamiana* leaves; consequently, red pigmentation was observed in all infected leaves (Figure 3b). RNA was extracted from the red parts of the infected leaves, and expression of the transgene was confirmed through reverse transcription (RT)‐PCR (Figure 3c). The red pigments from the infected leaves were extracted and analysed by high‐performance liquid chromatography (HPLC) and liquid chromatography (LC)‐MS. The red pigments from the leaves expressing *CqAmaSy1* included amaranthin (Figure 3d,e); however, it was absent from the leaves infected with the plasmids carrying the other two candidates (Figure 3c,d), indicating that only *CqAmaSy1* exhibited amaranthin‐synthesizing activity.

Because quinoa is a heterotetraploid, it is highly likely that homologous genes exist, and a search for *CqAmaSy1* homologs resulted in the discovery of *CqAmaSy2*. Expression plasmids for this gene were constructed (Figure S3) and introduced into *Agrobacterium*. Transformed *Agrobacterium* harbouring an overexpression vector of *CqAmaSy2* with *CqCYP76AD1‐1*,* CqDODA‐1* and *CqCDOPA5GT* were co‐infiltrated into the leaves of *N. benthamiana*, and HPLC analysis revealed that the red pigments in the leaves included amaranthin (Figure 3b,d). Unlike *CqAmaSy1*,* CqAmaSy2* was not expressed in the quinoa hypocotyl (Figure 3a). Taken together, these results demonstrate that at least three genes in the quinoa genome (*CqAmaSy1a*,* CqAmaSy1b and CqAmaSy2*) encode proteins with amaranthin synthetase activity. In addition, *CqAmaSy1b* may be involved in amaranthin biosynthesis in the quinoa hypocotyl. In subsequent experiments, *CqAmaSy1* expressed in the hypocotyl was used in addition to betalain biosynthesis genes (*CqSYP76AD1‐1*,* CqDODA‐1* and *CqCDOPA5GT*) expressed in the hypocotyl.

### Identification of amaranthin synthetase in beet and amaranth

**Figure 4:**
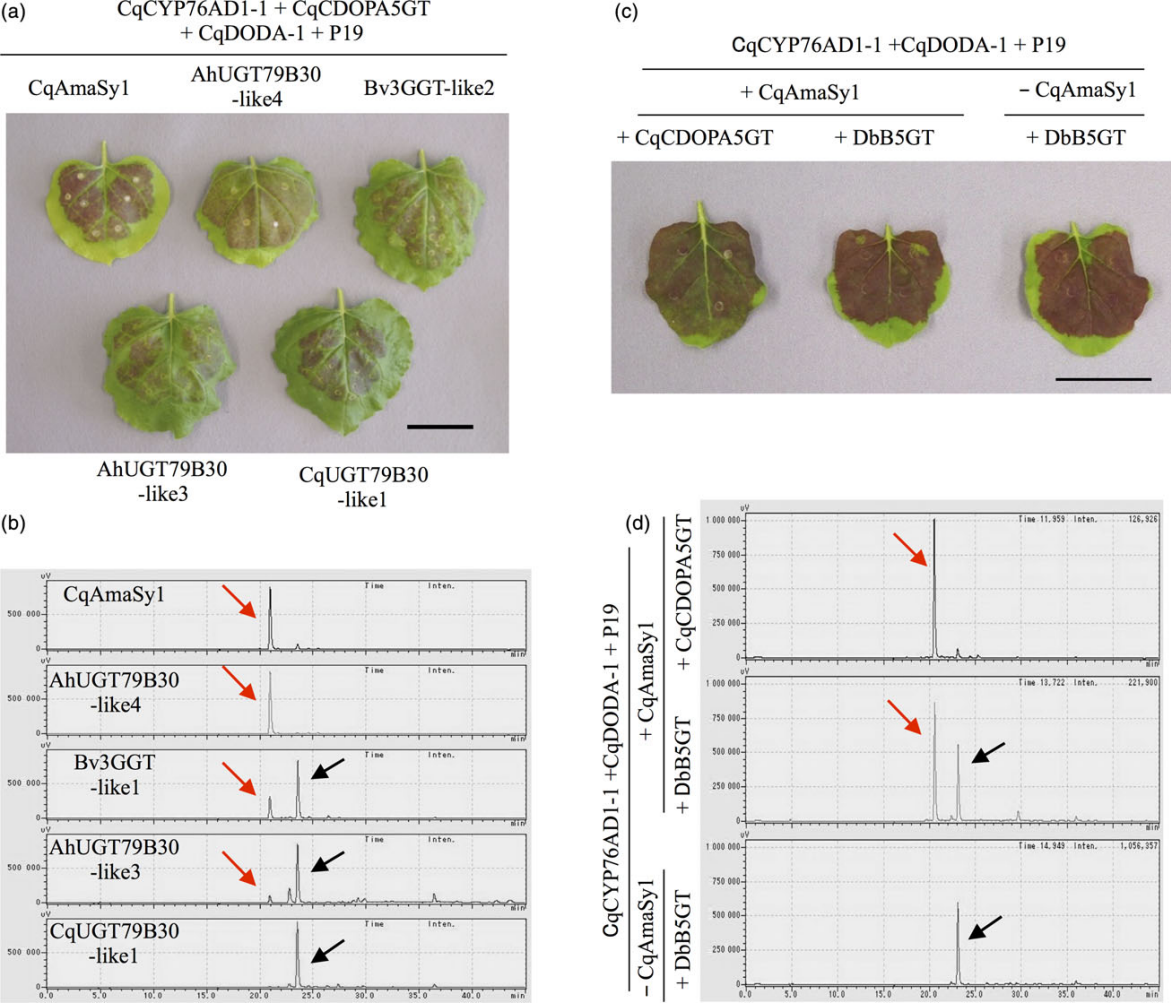
Identification of amaranthin synthetase in beets and amaranth and estimation of the substrate of CqAmaSy. (a) Recombinant expression of candidate amaranthin synthetase genes (*Bv3GGT‐like1*,* AhUGT79B30‐like3* and *AhUGT79B30‐like4*) and *CqAmaSy1* in *Nicotiana benthamiana* leaves. Co‐infiltration of transgenic *Agrobacterium* harbouring plasmids containing the candidate genes with *CqCYP76AD1‐1, CqCDOPA5GT*,* CqDODA‐1* and *P19*. CqUGT79B30‐like1 is the closest homolog of the amaranthin synthetase cluster. (b) HPLC chromatogram of infected *N. benthamiana* leaf extract. (c) Recombinant expression of *CqCDOPA5GT* or *DbB5GT* in *N. benthamiana* leaves. Co‐infiltration of transgenic *Agrobacterium* carrying plasmids for the expression of *CqCDOPA5GT* or *DbB5GT* with *CqCYP76AD1‐1, CqDODA‐1*,* CqAmaSy1* and *P19*. ‐ CqAmaSy1 served as the negative control. (d) HPLC chromatogram of the infected *N. benthamiana* leaf extract. Red and black arrows indicate amaranthin and betanin respectively. The horizontal axis indicates the retention time (min) and the vertical axis indicates the signal intensity (μV). Bars = 4 cm.

Although amaranthin synthetase was isolated from quinoa, we also investigated whether it exists in beets and amaranth, which also belong to the family Amaranthaceae. Phylogenetic analysis revealed the existence of one (Bv3GGT‐like1) and three (AhUGT79B30‐like2, AhUGT79B30‐like3 and AhUGT79B30‐like4) proteins similar to CqAmaSy1 and CqAmaSy2 respectively (Figure 2). Of these, plasmids that expressed *Bv3GGT‐like1*,* AhUGT79B30‐like3* and *AhUGT79B30‐like4* were constructed (Figure S4) and individually introduced into *Agrobacterium*; amaranthin synthetase activity was evaluated as described above. The presence of amaranthin was confirmed in all infected leaves (Figure 4a), and the leaves expressing *AhUGT79B30‐like4*, which is the closest orthologue of CqAmaSy, accumulated only amaranthin (Figure 4b). In contrast, leaves expressing *Bv3GGT‐like1* or *AhUGT79B30‐like3* accumulated both amaranthin and betanin (Figure 4b). These results demonstrate that amaranthin synthetase is also present in amaranthaceous plants other than quinoa.

Structural analysis was performed on the proteins used for phylogenetic tree analysis; consequently, the amino acid residues involved in enzyme activity were found to be conserved in all proteins, but those involved in amaranthin biosynthesis could not be identified (Figures S5 and S6).

### Determination of the substrate of CqAmaSy

We revealed that CqAmaSy has the ability to synthesize amaranthin. However, it remains unknown whether the reaction substrate of CqAmaSy is betanin (pathway I) or cyclo‐DOPA 5‐*O‐*glucoside (pathway II; Figure 1a). Therefore, we evaluated which substrate is catalysed by CqAmaSy using the *N. benthamiana* transient expression system. For evaluation of the CqAmaSy substrate, the use of *CqCDOPA5GT* in this expression system is inappropriate, because CqCDOPA5GT synthesize both betanin and cyclo‐DOPA 5‐*O‐*glucoside. Therefore, *betanidin‐5GT* derived from *Dorotheanthus bellidiformis* (*DbB5GT*, Figure 1a, Vogt *et al*., 1999) was used instead of *CqCDOPA5GT* since the former produces only betanin. A plasmid‐expressing *DbB5GT* was constructed (Figure S4) and introduced into *Agrobacterium*. Transformed *Agrobacterium* harbouring an overexpression vectors for *DbB5GT*,* CqCYP76AD1‐1*,* CqDODA‐1* and *CqAmaSy1* were co‐infiltrated into *N. benthamiana* leaves. The presence of amaranthin was confirmed in infected leaves (Figure 4c,d). This result indicated that *CqAmaSy* synthesizes amaranthin using betanin as a substrate.

### Production of betacyanin pigments in BY‐2 cells

**Figure 5:**
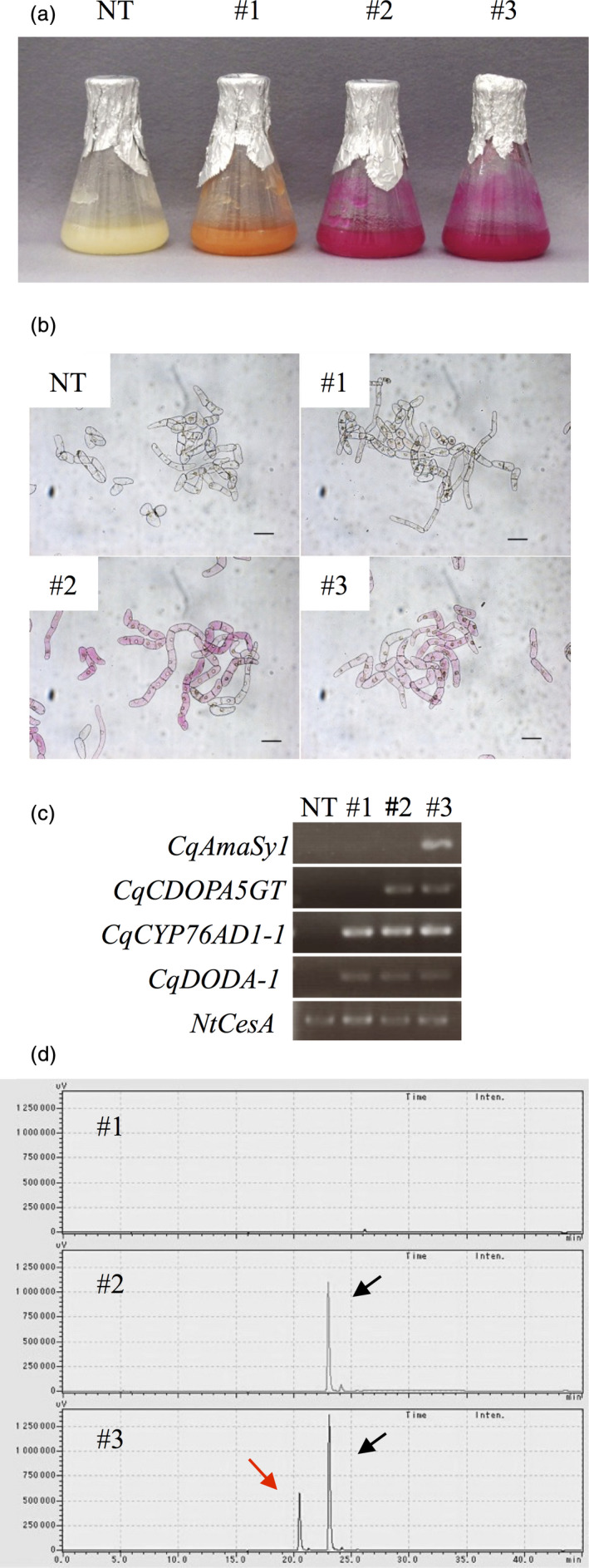
Production of betalain pigments in BY‐2 cells. (a) Photographs of the transformed BY‐2 cell lines at 2 weeks after transplantation. #1, #2 and #3 indicate transgenic BY‐2 cell lines producing betanidin, betanin and amaranthin respectively. NT denotes the non‐transgenic BY‐2 cell line. (b) Photographs of transformed BY‐2 cells. Bars = 100 μm. (c) RT‐PCR analysis of gene expression in transformed BY‐2 cells. *NtCesA* served as the internal control. (d) HPLC chromatograms of extracts of the transformed BY‐2 cell lines. Red and black arrows indicate amaranthin and betanin respectively. The horizontal axis indicates the retention time (min) and the vertical axis indicates the signal intensity (μV).

We identified the genes necessary to synthesize amaranthin (*CqCYP76AD1*,* CqDODA*,* CqCDOPA5GT* and *CqAmaSy*) in quinoa. We therefore introduced these genes into BY‐2 cells and attempted to mass produce betacyanin pigments. Plasmids carrying different drug resistance genes were first constructed to overexpress *CqCYP76AD1‐1*,* CqDODA‐1* and *CqCDOPA5GT* (Figure S7). The transformed *Agrobacterium* harbouring these plasmids was prepared and introduced into the BY‐2 cells. A betanidin‐producing cell line harbouring *CqCYP76AD1‐1* and *CqDODA‐1*, a betanin‐producing cell line harbouring *CqCYP76AD1‐1*,* CqDODA‐1* and *CqCDOPA5GT*, and an amaranthin‐producing cell line harbouring *CqCYP76AD1‐1*,* CqDODA‐1*,* CqCDOPA5GT* and *CqAmaSy1* were established (Figure 5a,b). After confirming transgene expression by RT‐PCR (Figure 5c), a cell line exhibiting intense coloration was selected and grown in a liquid culture. The betanin‐ and amaranthin‐producing cell lines were a vivid red, whereas the betanidin‐producing cell line was orange and never changed to red during the course of this study (Figure 5a). Pigments were extracted from these cell lines and analysed using HPLC. Only betanin was produced in the betanin‐producing cell line, whereas both betanin and amaranthin were produced in the amaranthin‐producing cells (Figure 5d). Betanidin production was barely detectable in the betanidin‐producing cell line. The betanidin‐producing cell line generated concentrations (mean ± SEM) of 19.53 ± 8.60 μm of betanin, whereas, the amaranthin‐producing cell line generated 13.67 ± 4.13 μm amaranthin and 26.60 ± 1.53 μm betanin. Almost no isobetanin or isoamaranthin was detected in the betanin‐ and amaranthin‐producing cell lines (Figure 5d).

### Effect of betanin and amaranthin on cell proliferation of human breast cancer cells

**Figure 6:**
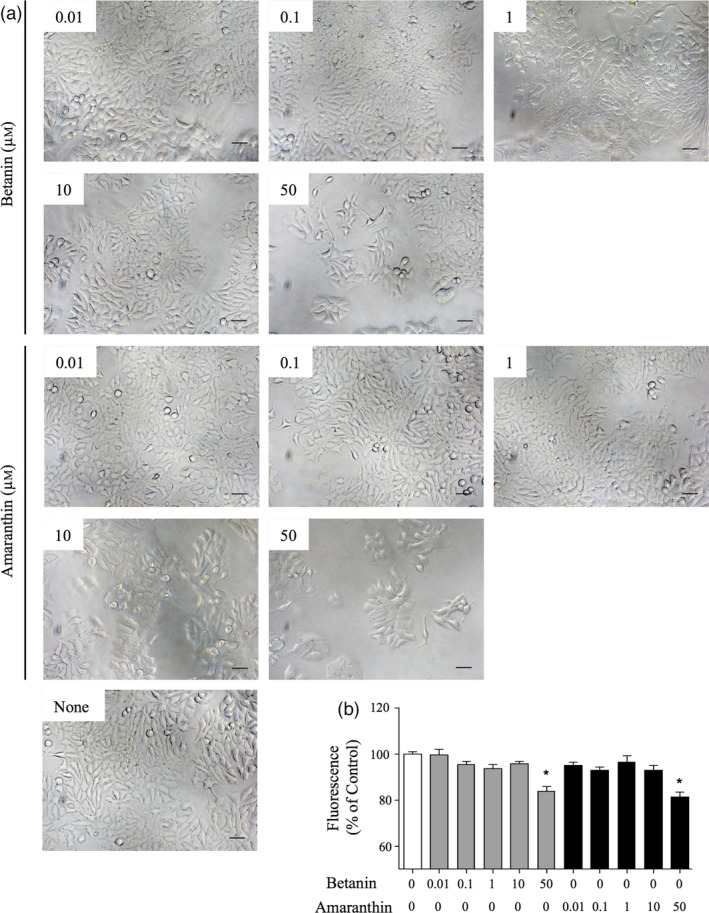
Evaluation of cell proliferation of MCF‐7 cells. (a) Morphology of MCF‐7 cells cultured for 72 h with betanin or amaranthin. Cells were examined under an inverted microscope. Bars = 100 μm. (b) MCF‐7 cells were cultured with increasing concentrations of betanin or amaranthin (0.01, 0.1, 1, 10 and 50 μm) for 72 h. Cell proliferation was determined using an AlamarBlue assay according to the manufacturer's instructions. Bars represent the mean ± SEM (*n* = 5); *P < 0.05 compared with untreated cells.

A previous report demonstrated that betanin‐enriched red beetroot extract induced the death of MCF‐7 cells (Nowacki *et al*., 2015); we therefore purified betanin and amaranthin and monitored their effects on the proliferation of MCF‐7 cells in culture. Both compounds markedly reduced the frequencies of altered cell morphologies and spreading (Figure 6a). Both compounds also slightly but significantly suppressed cell proliferation at concentrations of 50 μm (Figure 6b).

### Evaluation of amaranthin as an HIV‐1 protease inhibitor

**Figure 7:**
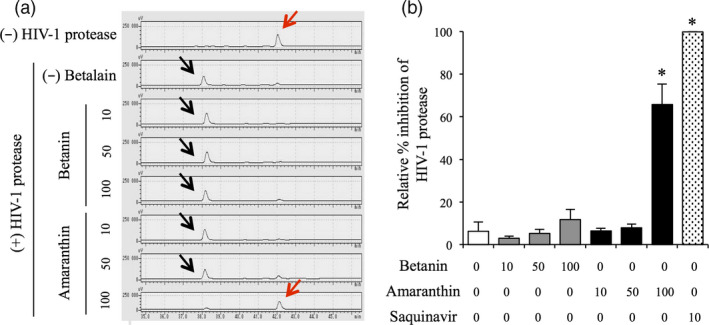
Evaluation of the inhibition of HIV‐1 protease. (a) HPLC chromatogram of the HIV‐1 protease reaction mixture. Red and black arrows indicate HIV‐1 protease substrate and its degradation product respectively. (+) and (−) HIV‐1 protease indicate the reaction mixture with or without HIV‐1 protease respectively. (−) betanin indicates the reaction mixture without betalain pigments. The horizontal axis indicates the retention time (min) and the vertical axis indicates the signal intensity (μV). (b) Relative amount of HIV‐1 substrate in reaction mixture. Grey, black and dotted bars indicate reaction mixtures containing betanin, amaranthin and saquinavir respectively. White bar indicates reaction mixture without betalain pigments as a negative control. Bars represent the mean ± SEM (*n* = 3); *P < 0.05 compared with the reaction mixture without betalain pigment; 0, 10, 50 and 100 indicate 0, 10‐, 50‐ and 100‐fold molar excess of betalain over HIV‐1 protease respectively.

New HIV‐1 protease inhibitor candidates were recently predicted using virtual screening of plant‐derived small molecule libraries, and amaranthin was listed as the third best of all the candidates (Yanuar *et al*., 2014); however, because this method was not empirically verified, we directly tested whether amaranthin inhibits HIV‐1 protease activity. Amaranthin and betanin were extracted and purified from the transformed BY‐2 cell line and added to a mixture of HIV‐1 protease, and HIV‐1 protease substrate, whose cleavage was monitored by HPLC. Intact HIV‐1 protease substrate was not detected in the presence of 10‐ and 50‐fold betanin and 10‐fold amaranthin (Figure 7). In 100‐fold betanin and 50‐fold amaranth, no significant difference was found between the controls, but a slightly intact HIV‐1 protease substrate could be detected (Figure 7). On the other hand, in 100‐fold amaranthin, it was possible to detect a predominantly intact HIV‐1 protease substrate (Figures 7 and S8). These results indicated that amaranthin indeed inhibits HIV‐1 protease, but betanin exhibits a weaker inhibition than amaranthin. The HIV‐1 protease inhibitory activity of amaranthin was also significantly lower than that of saquinavir, an artificial HIV‐1 protease inhibitor.

## Discussion

In this study, we succeeded in isolating amaranthin synthetase from quinoa by virtue of its homology to the amino acid sequences of various flavonoid glycosyltransferases that form β‐1,2‐glucosidic bonds. Amaranthin synthetase is considered to be a betalain‐modifying enzyme that is common to the family Amaranthaceae, and its orthologues are present in both beets and amaranth. Amaranthin is biosynthesized through two pathways (Figure 1), but transient expression in *N. benthamiana* revealed that CqAmaSy synthesizes amaranthin using betanin as a substrate (Figure 4d). Previous studies have also reported that cyclo‐DOPA 5‐*O*‐glucoside is an effective precursor for amaranthin biosynthesis (Sciuto *et al*., 1974), and *CqCDOPA5GT*, which encodes an enzyme which synthesizes this intermediate, is expressed in the quinoa hypocotyl. Based on these findings, amaranthin biosynthesis in quinoa is likely to involve the production of betanin from cyclo‐DOPA 5‐*O*‐glucoside followed by the synthesis of amaranthin from betanin by CqAmaSy. In the hypocotyl, quinoa, beet and amaranth accumulated different betalain pigments (Figure 1a,b). Betanin accumulated only in beet hypocotyl. It is considered that amaranthin synthetases such as Bv3GGT‐like1 do not function in the beet hypocotyl. In the hypocotyls of quinoa and amaranth, amaranthin is synthesized from betanin by the amaranthin synthetase (CqAmaSy1 and AhUGT79B30‐like4). In addition, in quinoa hypocotyls, it is expected that celosianin II is synthesized by amaranthin feruloyltransferase (unidentified) which adds ferulate to amaranthin. Differences between plant species in terms of the accumulation of betalain pigments in hypocotyls are presumed to be caused by differences in the action of these betalain‐modifying enzymes. Betalains are known to be involved in plant responses to environmental stress (Jain *et al*., 2015). The hypocotyl must be protected in order for plants to endure environmental stress. Thus, the composition of betalain pigments accumulating in the hypocotyl might be related to plant tolerance to environmental stress. In this study, we illustrated that CqAmaSy1 may be involved in amaranthin biosynthesis in the quinoa hypocotyl, and a more extensive analysis of CqAmaSy1 should help to clarify the physiological function of betalain pigments in these organs.

This study succeeded in artificially synthesizing amaranthin for the first time. Previously, amaranthin could only be extracted from plants such as amaranth and quinoa; however, establishment of this new production system now makes it possible to produce amaranthin on demand. Another advantage of amaranthin production using non‐betalain‐producing plants is that target substances can be produced by combining specific betalain biosynthesis genes. Furthermore, purification will be simplified because fewer types of betalain are produced when compared with the number present in extracts of betalain‐producing plants. The transient expression system developed in this study using *N. benthamiana* leaves produced mostly amaranthin, whereas both amaranthin and betanin were produced during constitutive gene expression in BY‐2 cells. This difference could be the result of differences in the two expression systems. One possible reason is that the expression level of CqAmaSy1 was lower in the constitutive expression system in BY‐2 cells, in which cell proliferation was active, than in *N. benthamiana* leaves. No betanidin accumulation could be detect in the betanidin‐producing cell line despite the expression of the two transgenes (*CqCYP76AD1‐1* and *CqDODA‐1*). This result may be because betanidin is labile compared with other betacyanins (Gandia‐Herrero *et al*., 2007; Grewal *et al*., 2018; Wybraniec *et al*., 2011).

We evaluated the effects of purified amaranthin and betanin derived from a transformed BY‐2 cell line on a human breast cancer cell line (MCF‐7). Both amaranthin and betanin slightly but significantly suppressed MCF‐7 cell viability, revealing a new biological effect of both compounds (Figure 6). It had been reported that betanin/isobetanin concentrate induces apoptosis and autophagic cell death of MCF‐7 cells (Nowacki *et al*., 2015). It might be expected that amaranthin and betanin would suppress the viability of MCF‐7 cells by inducing apoptosis and autophagic cell death similar to that induced by the betanin/isobetanin concentrate.

In addition, we demonstrated that amaranthin functions as an HIV‐1 protease inhibitor (Figure 7). Inhibition of HIV‐1 protease activity by amaranthin was dose‐dependent. On the other hand, betanin showed weaker inhibition of this enzyme than that by amaranthin (Figure 7). These results suggested that the addition of one glucuronic acid moiety is important for HIV‐1 protease inhibition. Amaranthin was previously selected as the third most effective potential inhibitor of HIV‐1 protease activity through virtual screening of substances that bind to the binding site of saquinavir, an artificial HIV‐1 protease inhibitor (Yanuar *et al*., 2014). This suggests that amaranthin binds to HIV‐1 protease and inhibits its function through the same mechanism as saquinavir. Other plant extracts and substances isolated from intact plants that inhibit HIV‐1 protease activity have been reported (Filho *et al*., 2010; Fujioka *et al*., 1994). This study demonstrated that amaranthin can be produced in suspension‐cultured cells from non‐betalain‐producing plants, which suggests that other pharmaceutically active compounds could be similarly produced. Some drugs derived from plant cells have already been approved by the USFDA (Yusibov *et al*., 2011); thus, in the future, betacyanin (and especially amaranthin) derived from cultured plant cells could become a source of large quantities of inexpensive pharmaceutical agents.

In this study, we identified and isolated amaranthin synthetase and succeeded in producing the betalain pigment amaranthin in BY‐2 cells. Furthermore, we identified a new bioactivity of amaranthin. Since it appears that individual betalain pigments possess their own unique biological activities, this research will enable the mass production of a variety of betalains in the future and contribute to the discovery of new biological effects of these pigments.

## Experimental procedures

### Plant materials and growth conditions

Seeds of the CQ127 variety of quinoa were obtained from the U.S. Department of Agriculture. Seeds of the 4099 variety of amaranth were obtained from the National Agriculture and Food Research Organization. Seed of beet cultivars Detroit Dark Red was purchased from Takii Seed Co., Kyoto, Japan. Quinoa, beet and amaranth seeds were sown in a cell tray and were grown at 23 °C with a 12‐h light/12‐h dark photoperiod in a phytotron.

Tobacco BY‐2 cells were grown in Linsmaier and Skoog medium supplemented with 3% sucrose and 0.2 mg/L 2,4‐dichlorophenoxyacetic acid at 26 °C (Nagata *et al*., 1992).

### Phylogenetic tree of deduced amino acid sequences

The ClustalW algorithm was used to align the deduced amino acid sequences of *Arabidopsis* flavonoid 3‐*O*‐glucoside 2‐*O*‐glucosyltransferase protein (UGT79B6, NP_200212) with flavonoid glycosyltransferase homologs from other plant species (Table S1, Thompson *et al*., 1994). The neighbour‐joining algorithm of MEGA7 software was used to construct a phylogenetic tree (Kumar *et al*., 2016).

### Molecular cloning

Total RNA was extracted using an RNeasy Plant Mini kit (Qiagen, Valencia, CA) and was treated with RNase‐free DNase I (Qiagen) to eliminate genomic DNA. A TaKaRa RNA PCR kit (AMV) Ver. 3.0 (TaKaRa, Kusatsu, Japan) with oligo(dT) primers was used to synthesize first‐strand cDNA from 500 ng total RNA. We obtained the full‐length ORF sequences of *CqAmaSy1b, CqAmaSy2* (XM_021880149)*, Cq3GGT, CqUGT79B30‐like1* and *Bv3GGT‐like1* from the NCBI gene database. We obtained the full‐length ORF sequences of *AhUGT79B30‐like4* and *AhUGT79B30‐like3* from the Phytozome gene database. *AcGFP1* was used as the vector control (TaKaRa).

### RT‐PCR analysis

A High Capacity cDNA Reverse Transcription kit (Thermo Fisher Scientific, Waltham, MA) with random primers was used to synthesize first‐strand cDNA from 500 ng of total RNA. A GeneAtlas 322 (Astec, Fukuoka, Japan) with PrimeSTAR GXL DNA Polymerase (TaKaRa) was used to perform RT‐PCR. The procedure for amplification of the candidate transcripts comprised initial denaturation at 94 °C for 2 min, followed by 35 cycles at 98 °C for 10 s, 55 °C for 15 s and 68 °C for 1.5 min. *CqCYP76AD1‐1* was used as a positive control for quinoa hypocotyl expression (Imamura *et al*., 2018). *L23* and *NtCesA* were used as internal controls for the expression in *N. benthamiana* leaves and tobacco BY‐2 cells respectively (Grimberg *et al*., 2015; Nakagawa and Sakurai, 2001). Primer pairs are listed in Table S2.

### Plasmid construction

PrimeSTAR GXL DNA polymerase and oligonucleotides containing a restriction enzyme cleavage site were used to perform PCR amplification (Table S2). In the agro‐infiltration analysis using *N. benthamiana*, the amplified fragments of candidate genes involved in amaranthin biosynthesis were digested with the appropriate restriction enzymes and then introduced into the binary vector pCAMBIA1301MdNcoI (Imamura *et al*., 2018). The other expression vectors (pCAM‐CYP76AD1‐1, pCAM‐CqDODA‐1, pCAM‐CqDODPA5GT and pCAM‐AcGFP1) were constructed in a previous study (Imamura *et al*., 2018). In the stable transformant analysis using BY‐2 cells, the amplified fragments of *CqCYP76AD1‐1* and *CqDODA‐1* were digested using the appropriate restriction enzymes and introduced into the binary vector pBI121. The amplified fragments of *CqDODPA5GT* introduced into the binary vector pBICBP35S, which harboured the bialaphos herbicide‐resistance gene, *bar* (Mori *et al*., 1993), were cut using *Stu*I, which is located downstream of the CaMV35S promoter. BigDye terminator chemistry and an ABI PRISM 3100 genetic analyser (Applied Biosystems, Foster City, CA) were used to sequence the resulting plasmids.

### Transient expression in *N. benthamiana*

Expression constructs were transformed into *Agrobacterium tumefaciens* strain GV3101 using the triparental mating method (Wise *et al*., 2006). The transformed *Agrobacterium* suspensions were infiltrated into the leaves of 5‐ to 6‐week‐old *N. benthamiana* plants, as described previously (Shamloul *et al*., 2014). The infiltrated plants were cultivated in a growth chamber at 23 °C and 60% humidity under long day conditions (16‐h light/8‐h dark).

### Stereostructural analysis

Three‐dimensional structures of the proteins were modelled on the Phyre2 web server (Kelley *et al*., 2015). UDP‐glucosyltransferase (PDB code; 5NLM) was selected as a template for homology modelling; 97% of amino acid residues were involved in the modelling.

### Transformation of BY‐2 cells

Tobacco BY‐2 cells were grown in Linsmaier and Skoog medium supplemented with 3% sucrose and 0.2 mg/L 2,4‐dichlorophenoxyacetic acid at 26 °C (Nagata *et al*., 1992). The *A. tumefaciens* strain GV3101, which harbours a Ti plasmid, was used to transform the cells as described previously (Hagiwara *et al*., 2003). Transgenic lines were selected on agar medium containing the appropriate selective agents, namely 50 mg/L hygromycin, 25 mg/L bialaphos or 100 mg/mL kanamycin with 500 mg/L carbenicillin. Suspension cells developed from calli were grown in 3 mL liquid medium in six‐well culture plates during primary screening, after which they were transferred to 150 mL liquid medium in 500‐mL flasks with constant shaking at 135 rpm. After initial culture for 2–3 weeks, the suspension cells were maintained without selective agents.

Cells were examined using an Axiovert 200 optical microscope (Zeiss, Jena, Germany), and images were captured using Axiovision 4.6 software (Zeiss).

### Plant pigment chemical analysis

Pigments were extracted from *N. benthamiana* leaves and BY‐2 cells and analysed as described previously (Hatlestad *et al*., 2012) and extracts were concentrated using a centrifugal concentrator (CC‐105, Tomy Seiko Inc., Tokyo, Japan).

A Shimadzu LC‐20AD system (Kyoto, Japan) was used for analytical HPLC separations. Samples were separated on a Shim‐pack GWS C18 column (5 μm; 200 × 4.6 mm i.d.; Shimadzu GLC, Tokyo, Japan), and linear gradients were run from 0% B to 45% B over 45 min using 0.05% trifluoroacetic acid (TFA) in water (solvent A) and 0.05% TFA in acetonitrile (solvent B) at a flow rate of 0.5 mL/min at 25 °C, with elution being monitored by absorbance at 536 nm. For evaluation of the biological activities of betalains, amaranthin‐ or betanin‐containing HPLC fractions were collected, evaporated to dryness, and the residues were dissolved in water and stored at −20 °C until needed.

### LC‐MS analysis

A Shimadzu LC‐20AD system equipped with an electrospray ionization Fourier transform ion cyclotron resonance mass spectrometer (Solarix, Bruker Daltonics, Billerica, MA) operating in the positive mode was used to perform the LC‐MS analysis. An XBridge C18 column (150 × 2.1 mm) with a 3.5‐μm particle size (Waters) was used for the separation. The flow rate was 0.3 mL/min using 0.1% TFA in acetonitrile. A stepwise gradient was employed using 0%, 10%, 50% and 100% acetonitrile at 0–3, 3–15, 15–20 and 20–25 min respectively.

### UV–Vis spectroscopy

A UV‐2450 (Shimadzu) spectrophotometer was used for UV–Vis spectroscopy. Betalain pigment concentration was determined using molar extinction coefficients of ε = 54 000 m/cm at 536 nm for betanidin and 65 000 m/cm at 536 nm for betanin and amaranthin (Gandía‐Herrero *et al*., 2010; Schwartz and Von Elbe, 1980). All measurements were performed at 25 °C.

### Determination of cell viability of human breast cancer cells

MCF‐7 cells (human breast cancer), which were obtained from the RIKEN BioResource Center (Ibaraki, Japan), were cultured in Dulbecco's modified Eagle's medium‐high glucose (4.5 g/L glucose; DMEM‐HG) supplemented with 10% foetal bovine serum (FBS) and antibiotics (100 U/mL penicillin and 100 μg/mL streptomycin). Cells were maintained at 37 °C in a 5% CO_2_/95% air atmosphere at 100% humidity. Cells seeded at 5000 cells/well on 96‐well plates were cultured with increasing concentrations of betanin and amaranthin for 72 h. Cell proliferation was monitored using an AlamarBlue assay (Thermo Fisher Scientific) according to the manufacturer's instructions. Fluorescence was detected with excitation at 560 nm and emission at 590 nm using a plate reader (Tecan Infinite M200; TECAN, Mannedorf, Switzerland).

### Evaluation of HIV‐1 protease activity

HIV‐1 protease activity was evaluated as described previously (Boso *et al*., 2015). For external HIV‐1 protease‐processing reactions, 0.4 μL recombinant HIV‐1 protease (approximately 16 pmol, ab84117, Abcam, Cambridge, MA) and 4 μL of 1 mm HIV‐1 protease substrate (Lys‐Ala‐Arg‐Val‐Nle‐p‐nitro‐Phe‐Glu‐Ala‐Nle amide, Sigma‐Aldrich, St. Louis, MO) were incubated with/without betanin or amaranthin in 30‐μL reaction volumes using phosphate buffer (25 mm NaCl, 25 mm Na_2_HPO_4_, 1 mm dithiothreitol, pH 4.7) at 25 °C for 2 h. Betalains used in the reaction were added in 10‐, 50‐ and 100‐ fold molar excess over HIV‐1 protease. Saquinavir mesylate (Sigma‐Aldrich) was used as a positive control. After incubation, the HIV protease substrate was quantified using HPLC.

A Shimadzu LC‐20AD system was used for the analytical HPLC separations. Samples were separated on a Shim‐pack GWS C18 column (5 μm; 200 × 4.6 mm i.d.; Shimadzu GLC), and linear gradients were established from 0% B to 50% B over 50 min using 0.05% TFA in water (solvent A) and 0.05% TFA in acetonitrile (solvent B) at a flow rate of 0.5 mL/min and temperature of 25 °C, with elution of the HIV‐1 protease substrate and its degradation products being monitored by absorbance at 214 nm.

### Statistical analysis

Comparisons among some groups were performed using one‐way analysis of variance with Dunnett's post‐hoc testing. Statistical analyses were performed using GraphPad Prism ver. 5.02 statistical software (GraphPad Software Inc., San Diego, CA). All results are expressed as the mean ± SEM. Differences were considered to be statistically significant for *P* < 0.05.

## Accession numbers

*CqAmaSy1a,* XM_021898385; *CqAmaSy1b,* XM_021898386; *CqAmaSy2*, XM_021880149; *Cq3GGT‐like2,* XM_021880147; *CqUGT79B30‐like1,* XM_021879979; *CqUGT79B6‐like1*, XM_021863575; *CqUGT79B6‐like2*, XM_021892276; *CqUGT79B6‐like3*, XM_021918046; CqUGT79B2‐like, XM_021875489; *Cq3GGT‐like1,* XM_021870862; *Cq3GGT‐like3,* XM_021910314; *CqUGT79B30‐like2,* XM_021880148; *CqUGT79B30‐like5,* XM_021902928; *CqCYP76AD1‐1*, XM_021913610; *CqDODA‐1*, XM_021913611; *CqCDOPA5GT*, XM_021892614; *Bv3GGT‐like1*, XM_010697515; *AhUGT79B30‐like2*, AH018627‐RA (Phytozome accessions); *AhUGT79B30‐like3*, AH018628‐RA (Phytozome accessions); *AhUGT79B30‐like4*, AH018629‐RA (Phytozome accessions); *betanidin‐5GT*, Y18871; and *UGT79B6*, NM_124780.

## Author contributions

HM and MM conceived this study. TI and MM designed the experiments. TI and MM designed and constructed the plasmids. TI conducted experiments to evaluate the amaranthin synthetase activity using *N. benthamiana*. MM constructed the amaranthin production system using BY‐2 cells. AM detected betalain pigments via LC‐MS analysis. YH conducted the cancer cell proliferation assay. SO conducted simulation analysis. MM, TI, NI and SO evaluated betalain pigments as HIV‐1 protease inhibitors. TI and MM wrote the manuscript. All authors have read and approved the final manuscript.

## Conflict of interest

The authors have no conflicts of interest to declare.

## Supporting information


**Figure S1** Schematic representation of the reaction for quercetin 3‐*O*‐beta‐glucosyl‐(1‐>2)‐beta‐glucoside (a), Amaranthin (b) and cyclo‐DOPA‐glucuronylglucoside (c).
**Figure S2** Expression analysis of *CqAmaSy1* in quinoa hypocotyls.
**Figure S3** Schematic representations of the plant expression vectors.
**Figure S4** Schematic representations of the plant expression vectors.
**Figure S5** Model structure of CqAmaSy1.
**Figure S6** Comparison of the deduced amino acid sequences of flavonoid 2″Gt 2″Rt 6″Rt cluster and the unknown cluster.
**Figure S7** Schematic representations of the plant expression vectors.
**Figure S8** Characterization of the HIV‐1 protease substrate.

**Table S1** Proteins used for phylogenetic analysis.
**Table S2** Primers used in this study.
