# Supplementary material for: Isolation of amaranthin synthetase from Chenopodium quinoa and construction of an amaranthin production system using suspension‐cultured tobacco BY‐2 cells
Source: Plant Biotechnol J. 2018 Dec 5;17(5):969–81. doi: 10.1111/pbi.13032 (PMC6587806; doi:10.1111/pbi.13032)
Supplement: Supplementary file 1 — Figure S1 Schematic representation of the reaction for quercetin 3‐O‐beta‐glucosyl‐(1‐>2)‐beta‐glucoside (a), Amaranthin (b) and cyclo‐DOPA‐glucuronylglucoside (c). Figure S2 Expression analysis of CqAmaSy1 in quinoa hypocotyls. Figure S3 Schematic representations of the plant expression vectors. Figure S4 Schematic representations of the plant expression vectors. Figure S5 Model structure of CqAmaSy1. Figure S6 Comparison of the deduced amino acid sequences of flavonoid 2″Gt 2″Rt 6″Rt cluster and the unknown cluster. Figure S7 Schematic representations of the plant expression vectors. Figure S8 Characterization of the HIV‐1 protease substrate. [file PBI-17-969-s001.pdf]

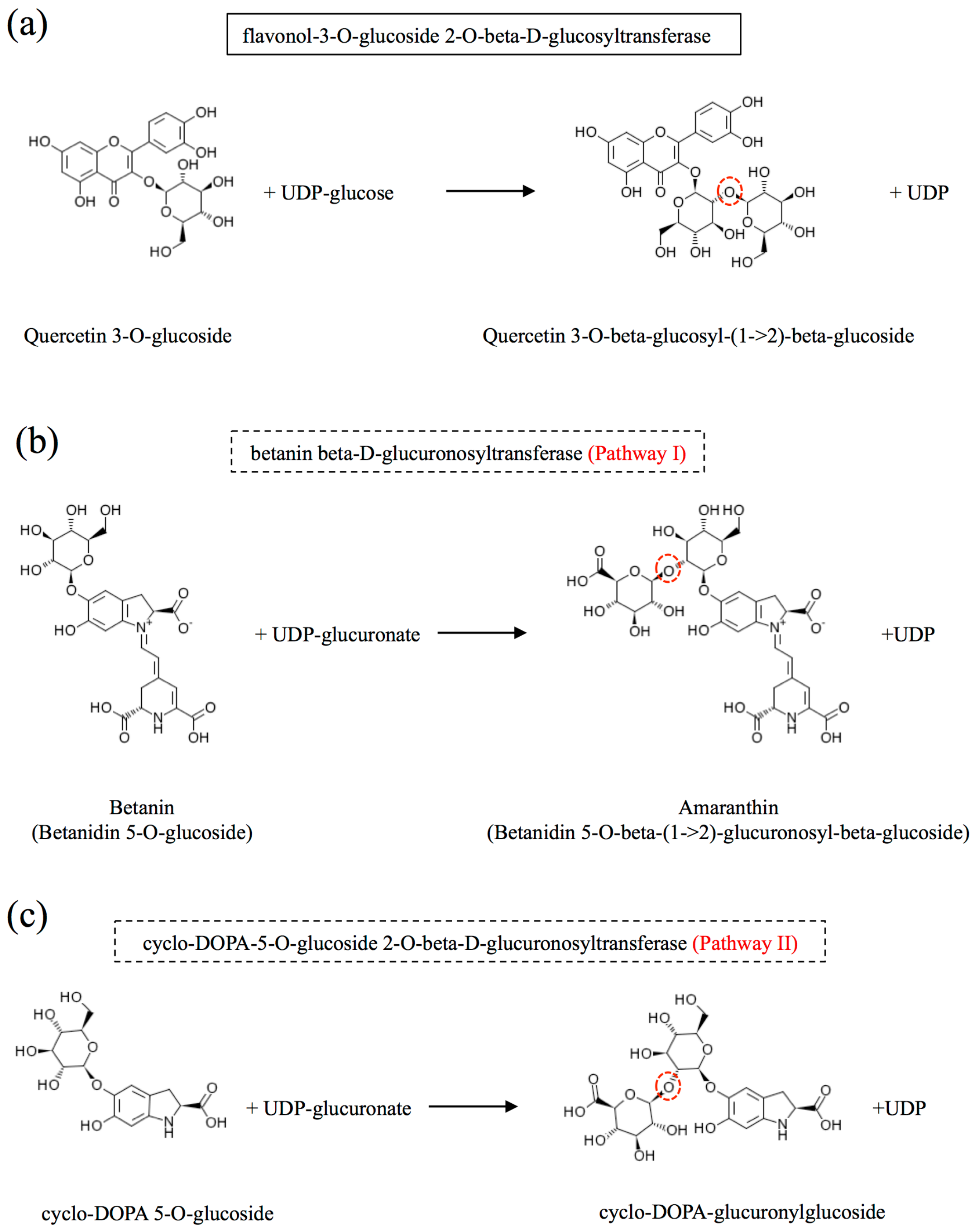

Figure S1 Schematic representation of the reaction for quercetin 3-*O*-beta-glucosyl-(1->2)-beta-glucoside (a), Amaranthin (b), and cyclo-Dopa-glucuronylglucoside (c). Box and dashed box indicate identified and predicted enzymes, respectively. Red-dashed circles indicate β-1,2-glycosidic bond.

(a)

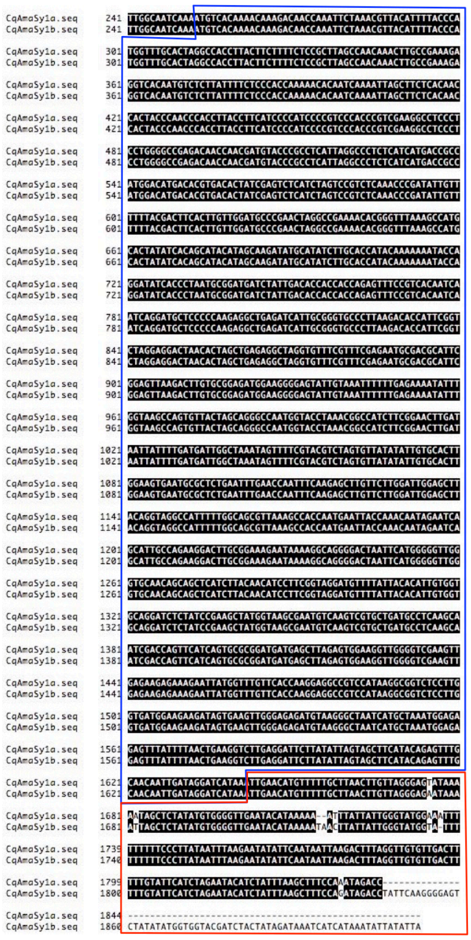

(b)

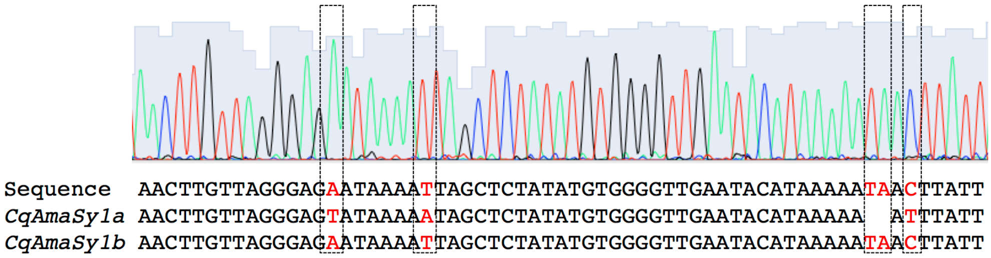

Figure S2 Expression analysis of *CqAmaSy1* in quinoa hypocotyls. (a) Alignment of the *CqAmaSy1* sequences. Upper and lower sequences indicate *CqAmaSy1a* (XM_021898385) and *CqAmaSy1b* (XM_021898386), respectively. Blue and red boxes indicate coding sequence (CDS) and 3' untranslated region (UTR) of *CqAmaSy1*, respectively. (b) Direct sequence analysis for 3' UTR of *CqAmaSy1* transcripts from quinoa hypocotyls. Red characters indicate SNPs between *CqAmaSy1a* and *CqAmaSy1b*.

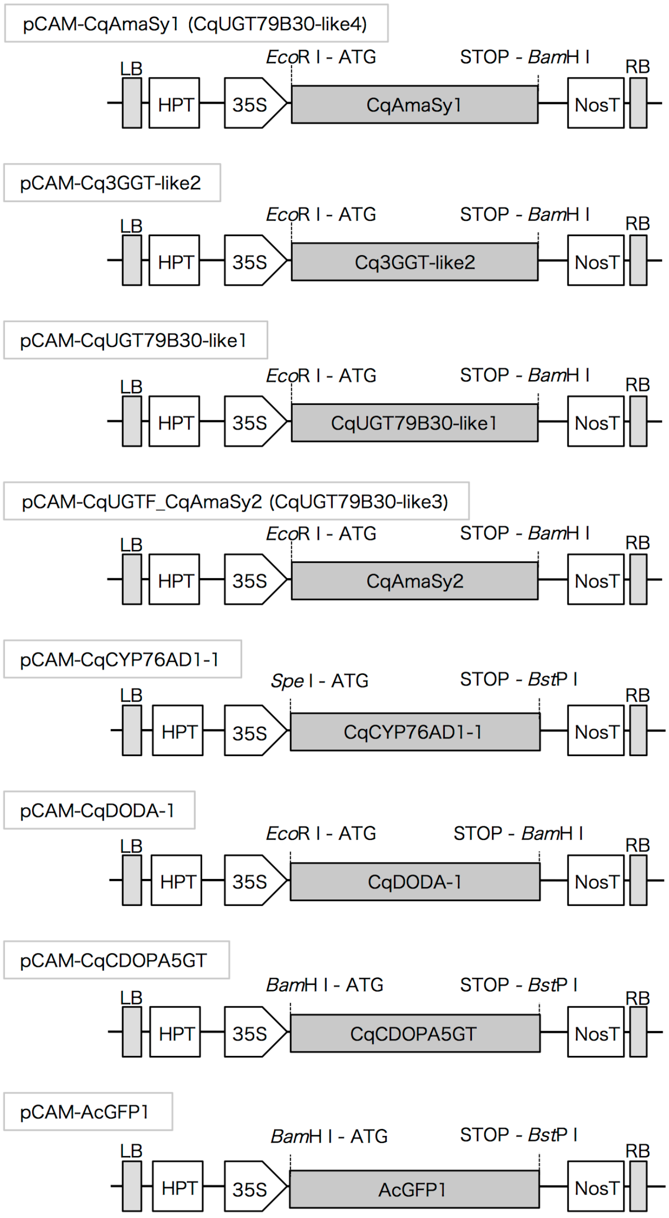

Figure S3. Schematic representations of the plant expression vectors. CqAmaSy1, *CqAmaSy1* CDS; CqAmaSy2, *CqAmaSy2* CDS; Cq3GGT-like2, *Cq3GGT-like2* CDS; CqUGT79B30-like1, *CqUGT79B30-like1* CDS; CqCYP76AD1-1, *CqCYP76AD1-1* CDS; CqDODA-1, *CqDODA-1* CDS; CqCDOPA5GT, *CqCDOPA5GT* CDS; AcGFP1, *AcGFP1* CDS; 35S, CaMV 35S promoter; NosT, *nopaline synthase* terminator; 35S-T, 35S terminator; RB, right border; LB, left border; HPT, *hygromycin phosphotransferase* expression cassette; ATG, start codon; and STOP, stop codon.

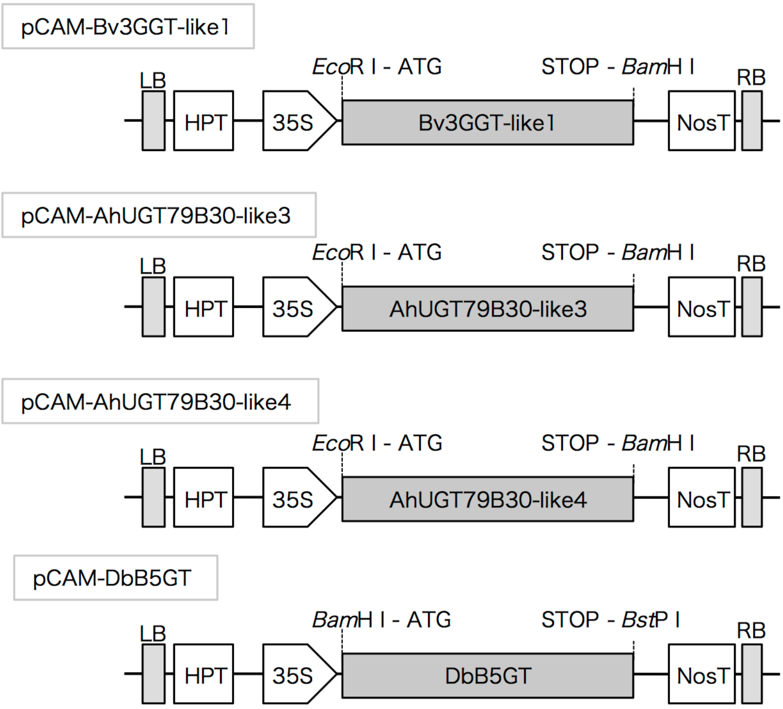

Figure S4 Schematic representations of the plant expression vectors. Bv3GGT-like1, *Bv3GGT-like1* CDS; AhUGT79B30-like3, *AhUGT79B30-like3* CDS; AhUGT79B30-like4, *AhUGT79B30-like4* CDS; DbB5GT, *DbBetanidin-5GT* CDS; 35S, CaMV 35S promoter; NosT, *nopaline synthase* terminator; 35S-T, 35S terminator; RB, right border; LB, left border; HPT, *hygromycin phosphotransferase* expression cassette; ATG, start codon; STOP, stop codon. Abbreviations for species: Ah, *Amaranthus hypochondriacus*; Bv, *Beta vulgaris*; *Db, Dorotheanthus bellidiformis.*

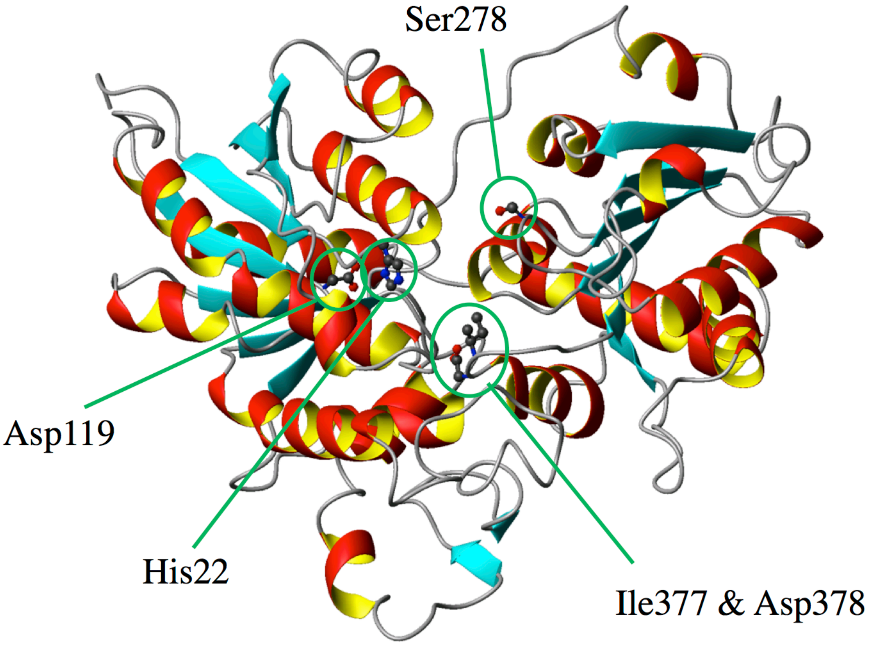

Figure S5 Model structure of CqAmaSy1. The structure was calculated using the coordinate UDP-glucosyltransferase (PDB code; 5NLM). The residues, expected to be responsible for the activity, are shown in ball-and-stick model.

```
6BK0:A|PDBID|CHAIN|SEQUENCE      -------------GMGSMSTPAASANGGQVLLLPFP-AAQGHTNPMLQFGRRLAYHGLRP      46
CqUGT79B2-like                   ------------------------MGGKQLKVLMYPWLAIGQIIPYLHLANKLAERGHIV      36
CqUGT79B6-like2                  MYATIQLHHPIINEIKKKKK--KNMKETQLKVLMHPWLAMGHFIPYLHLANKYAERGHIV      58
AtF3G2GT                         -------------------------MGSKFHAFMFPWFGFGHMTAFLHLANKLAEKDHKI      35
AhUGT79B6-like                   -------------------------MGKQLNLVMFPWFAIGHITPFLHLANKLAQNGHKT      35
BvUGT97B6-like1                  -------------------------MGKQLHIVMFPWFAIGHMTPFLHLSNKLAEYGHKI      35
BvUGT97B6-like2                  -------------------------MGKQLHIVMFPWFAIGHMTPFLHLSNKLAEYGHKI      35
CqUGT79B6-like1                  -------------------------MVKQLHIVMFPWFAVGHMTPFLHLANKLADRGHKI      35
CqUGT79B6-like3                  -------------------------MVKQLHIVMFPWFAVGHMTPFLHLANKLAERGHKI      35
IpA3G2Gt                         ----------------------MGSQATTYHMAMYPWFGVGHLTGFFRLANKLAGKGHRI      38
GmF3G2Gt                         ------------------------MKSRPLHIAMYPWLAMGHQIAFLHLCNKLAIRGHKI      36
CqUGT79B30-like5                 ------------MDKKIASM--VEEKERPLHIAMYSWFAFGHLTSFLQLANKLAERGHKV      46
BvUGT79B30-like                  -------------------M--AEKIEKPLHIAMYSWFAFGHLTSFLQLANKLAQRGHKI      39
Cq3GGT-like2                     ----------------MSSS--NNNNGKTLHMAMYPWFAFGHLTSFLHLANKLAERGHKV      42
CqUGT79B30-like2                 -----------------MSK--INETNECLHIAMYPWFAFGHLTYFLHLANKFAQRGHKI      41
AhUGT79B30-like1                 -------------------M--SKENNGNLHIAMWPWFAFGHITSFLHLANKLAEKGNKI      39
Cq3GGT-like1                     -----------MSKENGIAN--GNGNCKHLHIAMCPWYAFGHITSFLHLANKLAEKGHKI      47
Cq3GGT-like3                     -----------MSKENGIAN--GNGNCKHLHIAMCPWYAFGHITSFLHLANKLAEKGHKI      47
Bv3GGT-like2                     -----------------MSK--ENGNSEHLHIAMCPWFAFGHITSFLHLANKLAEKGHTI      41
CqUGT79B30-like1                 ------------------MS--NNKNSKILKVAFYPWYAFGHLIPNLRLANQLAERGHQI      40
AhUGT79B30-like4                 -----------------MSH--NKESNPNLHVAFYPWFALGHLTSFLRLANKLAERGLQV      41
CqAmaSy2                         -----------------MSQ--N-KDTQILNVAFYPWFALGHLTSFLRLANKLAERGHKV      40
CqAmaSy1                         -----------------MSQ--N-KDNQILNVTFYPWFALGHLTSFLRLANKLAERGHNV      40
Bv3GGT-like1                     ------------------MG--ENKESQVLKVAFYPWFALGHLTSFLRLANKLAERGHQV      40
AhUGT79B30-like3                 ------------------MG--YKEESSVMHIAFYPWLALGHITSFLRLANKLAQKGHTI      40
AhUGT79B30-like2                 ------------------MG--YKEESSVMHIAFYPWLALGHITSFLRLANKLAQKGHTI      40
                                                                       . *:    ::: .: *  .

6BK0:A|PDBID|CHAIN|SEQUENCE      TLVTTRYVLST-----TPPPGD---PFR---VAAISDGFDDASGMAALPDPGEYLRTLEA      95
CqUGT79B2-like                   TLLLQNKAKQLLQSKNYHPSFITLHTITIPQVHPLPPGTETASD---IPFHLQTHLATAL      93
CqUGT79B6-like2                  TLLLPNKAKNLLQSKNLHPSFITLHTITIPHVHPLPPGTETASD---IPFHLQTHLATAL      115
AtF3G2GT                         TFLLPKKARKQLESLNLFPDCIVFQTLTIPSVDGLPDGAETTSD---IPISLGSFLASAM      92
AhUGT79B6-like                   ILLVPTKAKIHLDTLNYHPSLVTLQPILVPHVEPLPPGTDTASD---IPIHLHTHLATAL      92
BvUGT97B6-like1                  TFLIPKKAKHQLEHLLLYPSLITLHPITIPHVHPLPPGIETASE---IPLHLNNHLATAL      92
BvUGT97B6-like2                  TFLLPKKAKHQLQHLLLYPSLITLHPITIPHVHPLPPGTETASE---IPLHLNNHLATAL      92
CqUGT79B6-like1                  TFFLPNKAKIQLQHLVLYPNLITLHPITVPHVHPLPFGAETASD---IHLNLTGHLATAL      92
CqUGT79B6-like3                  TFLLPNKAKLQLQHFVLYPNLITLHPITVPHVHPLPFGTETASD---IHINLNGHLATAF      92
IpA3G2Gt                         SFLIPKNTQSKLESFNLHPHLISFVPIVVPSIPGLPPGAETTSD---VPFPSTHLLMEAM      95
GmF3G2Gt                         SFITPPKAQAKLEPFNLHPNSITFVTINVPHVEGLPPDAQTTAD---VTYPLQPQIMTAM      93
CqUGT79B30-like5                 SFFLPNKTQPKLTPHNHHPHLITFIPITIPAVKGLPCEAETTAD---VPYEDRSLIMEAM      103
BvUGT79B30-like                  SFFLPTKTQQKLTPHNHHPHLITFIPIAVPAVEGLPSGAETTAD---VPYEARSLIMAAM      96
Cq3GGT-like2                     SFFLPTKTQTKLASHNRHPNLITFIPISVPSVHGLPAGAETTND---VSAQARPSLMDAM      99
CqUGT79B30-like2                 SFFLPTKTQPKLSIHNHHPNLITFIPINVPHVDGLPFGAETTND---VSSSSYPLIMDAM      98
AhUGT79B30-like1                 SFFLPTKTKLKFVSHNHHPHLIDLIPITVPAVDGLPIGAETTAD---VSDSNRHLIMSAI      96
Cq3GGT-like1                     SFFLPTNTKHRFTSHNHHPNLITLIPITVPAVDGLPVGAETTAD---VPASSRPLIMTAM      104
Cq3GGT-like3                     SFFLPTNTKHRFASHNHHPNLITLIPLTVPAVDGLPVGAETTAD---VPASSRPLIMAAM      104
Bv3GGT-like2                     SFFIPTKTKLKFVSHNHHPHLITLIPITLPSVHGLHVGAETTAD---VPASSRSLIMAAM      98
CqUGT79B30-like1                 SFFLPSKIQTKLASYNHHPDHISFIPIDIPHVDGLPPGAGAANN---VPASALTFLMRAM      97
AhUGT79B30-like4                 SYFIPSKTQPKLSPHNHHPNHLTFIPITVPHVDGLPPGAETTND---VPGSAVPLIMTAM      98
CqAmaSy2                         SYFIPPKTRSKLASHNHYPTHLTFIPIPVPPVEGLPPGAETTND---VPASSGPLIMNAM      97
CqAmaSy1                         SYFLPPKTQSKLASHNHYPTHLTFIPIPVPPVEGLPPGAETTND---VPASLGPLIMTAM      97
Bv3GGT-like1                     SYFLPKNTQLKLASQNHFPDHLTFIPITVPSVDGLPPGAETTND---VSLSGAHLIMAAM      97
AhUGT79B30-like3                 SFFIPTKTQSKLASQNHFPNHLTFVPVDIPSIDGLPPGAETTND---VSVSAAPLIMSAM      97
AhUGT79B30-like2                 SFFIPTKTQSKLASQNHFPNHLTFIPVDIPSIDGLPLGAE-TND---VSVSAAPLIMSAM      96
                                   .               *       .    :  :      :     :

6BK0:A|PDBID|CHAIN|SEQUENCE      HGARTLAELLLSEARAGRPARVLVYDPHLPWARRVAR-AAGVATAAFLSQPCAVDLIYG      153
CqUGT79B2-like                   DLTRVEFESILVR--L-QP-DLIFYDFTYWVPEAVVASGSQAKCVA-YYIVSTATMAFR      147
CqUGT79B6-like2                  DLTRVEFESILVS--F-QP-NLVVYDFTYWVPEAVAASGSQAKCVA-YSIVSAVAMAFT      169
AtF3G2GT                         DRTRIQVKEAVS---VGKP-DLIFFDFAHWIPEIAR--EYGVKSVN-FITISAACVAIS      144
AhUGT79B6-like                   DLTRPQVESIITGLDP-KP-DLLFYDLAYWIPDIAS--KFKIKTVC-YKVLSAASQAIA      146
BvUGT97B6-like1                  DLTRPEVESFIITLQP-KP-HVLFYDMAYWVPEIAS--KLQIKSVC-YNVVCAASLAIA      146
BvUGT97B6-like2                  DLTRLEVEYFIITLQP-KP-HLLFYDIAFWVPEIAS--KLQIKSIC-YNVVCAASLAIA      146
CqUGT79B6-like1                  DLTRPEVESVIIGLSP-KP-HLFFYDIAHWASEIAA--KLGIKSVN-YNVVCAASLAIA      146
CqUGT79B6-like3                  DLTRPEFESVIIGLTP-KP-HLFFYDIAHWASEVAA--RLRITSVN-YNVVCAASLAIA      146
IpA3G2Gt                         DKTQNDIEIILKD--L-KV-DVVFYDFTHWLPSLAR--KIGIKSVF-YSTISPLMHGYA      147
GmF3G2Gt                         DLTKDDIETLLTG--L-KP-DLVFYDFTHWMPALAK--RLGIKAVH-YCTASSVMIGYT      145
CqUGT79B30-like5                 DLTRDTIDSLLSE--L-KP-DLVFFDFTEWLPGLAR--KHGAKSVY-YATMFAVSGAYF      155
BvUGT79B30-like                  DLTQDTIESLLSQ--L-KP-DLVFFDFTEWLPNVAR--KHGVKSVY-YATLLAVSGAYF      148
Cq3GGT-like2                     DLTRPDIDSHLSH--L-KP-DYIFFDFTEWVPSVAR--KYSIKSIY-YYTSYMANMAYC      151
CqUGT79B30-like2                 NLTRPDIDSKLGL--L-KP-DFIFFDFTEWLPDMAR--KHSVKSVY-YSTMYAVSFAYL      150
AhUGT79B30-like1                 ERIPETIDTHLSN--L-KP-DFVFFDFVEWVPKIAR--KHQIKSVF-YSTVYLSTVAYL      148
Cq3GGT-like1                     DQTQDTIEFYLSS--L-KL-DFIFFDFTEWVPKIAR--KHQVKSLF-YSTVYLVTVAYL      156
Cq3GGT-like3                     DQTQDTIEFYLSS--L-KP-DFIFFDFTEWVPKLAR--KHQVKSLF-YSTVYLVTVAYL      156
Bv3GGT-like2                     DQTQDTIESHLAH--I-KP-DFIFFDFTEWIPKVAR--KHQIKSVF-YSTVYLVTVAYL      150
CqUGT79B30-like1                 DMTRDTMEDHLLH--V-KP-DIVFYDFTCWMPELAR--KHGIKAVY-FCQSYVMTFAYF      149
AhUGT79B30-like4                 DLTQDIIEAHLAQ--L-KP-NFVFYDFTYWIPKLGQ--KLGFKSIH-YFTAFISRYGYL      150
CqAmaSy2                         DMTRDTIETHLVR--L-KP-DIVFYDFTCWMPELAR--KHGFKAIH-YITAYIARYAYL      149
CqAmaSy1                         DMTRDTIESHLVR--L-KP-DIVFYDFTCWMPELGR--KHGFKAMH-YITAYIARYAYL      149
Bv3GGT-like1                     DMTRDTIDAHLAL--L-KP-DFVFYDFAYWMPQLAR--KHGIKSVH-YITGLIARYTLA      149
AhUGT79B30-like3                 DMTRDSIETQLVH--L-KP-DFVFYDFAYWMPELGK--KHGFKSVH-YITGYIARYAAF      149
AhUGT79B30-like2                 DMTRESIETQLVH--L-KP-DFVFYDFAYWMPELGK--KHGFKSVH-YITGYLTRYAAF      148
                                 .      .  :            .      *
```

```
6BK0:A|PDBID|CHAIN|SEQUENCE      EVCARRLALPVTPTDARGLYARGVL-------GVELGPDDVPPFVAAPELTPAFCEA---          203
CqUGT79B2-like                   MVPSRDPPKGRAVVEEDACVACAPPGYPSS--TIILEPKR--ML-GL---YMPFGEG---          196
CqUGT79B6-like2                  MVPSRDPPKDKAVVEEDACVANAPPGYPSS--TVIIDPRT--MIRSL---HVPFGEG---          219
AtF3G2GT                         FVPGRS--------QDD--LGSTPPGYPSS--KVLLRGHETNSLSFL---SYPFGDG---          186
AhUGT79B6-like                   FVPALKLPMDRPLSETD--LAHPPLGYPSS--NVVFRGYETKSLLFV---GFEFGSG---          196
BvUGT97B6-like1                  LVPALNVGRYQPFTEED--VAKPPPGYPSS--NVVFHGPEARSLLFL---GLEFGSG---          196
BvUGT97B6-like2                  LVPALNVGRDQPVTEED--VAKPPPGYPSS--NVVFHGPEARSLLFI---GLEFGSG---          196
CqUGT79B6-like1                  LVPARNLARDRPLTEED--VARPPPGYPSS--NVVLHGPEAHSLLFL---GEDYGSG---          196
CqUGT79B6-like3                  LVPARNLPRDRPLIEED--VARPPSGYPSS--NVVLHGPEARSLLFL---GTDFGSG---          196
IpA3G2Gt                         LSPERRVV-GKQLTEAD--MMKAPASFPDP--SIKLHAHEARGFTAR-T-VMKFGGD---          197
GmF3G2Gt                         LTPARFHQ-GTDLMESD--LMEPPEGYPDS--SIKLQTHEARVFAAK-R-KDTFGSN---          195
CqUGT79B30-like5                 LPRARAQPG-----GQD--LSRPPPGFPSSKSMITLQAHEARAVGYMVT-HMEFGGKG--          205
BvUGT79B30-like                  LPRARAQLG-----GRD--LTRPPPGFPPSKSMITLRDHEARAVGYAVT-QMEFGGPG--          198
Cq3GGT-like2                     NLQARNLPDKHKYTEAD--LMSPPPGFPCP--VIRMRAQEARTFAYM-C-QMVFGGG---          202
CqUGT79B30-like2                 SPMARKLPPKYCFKEAD--LVEPPPGFPCS--AITLRPREARELAYI-C-SIEFGG----          200
AhUGT79B30-like1                 LPQPRNLPI-NCLTEEE--LKQPPAGYPMP--SIRLQAHEARSLANS-S-KRGFGGEEKS          201
Cq3GGT-like1                     LPQPRNLPI-NSFSEEE--MKKPPPGFPLP--SVRLRAHEARSLAGA-S-EAEFGGSG--          207
Cq3GGT-like3                     LPQPRNLPI-NSFSEDE--MKKPPPGFPLP--SVRLRAHEAHSLAGA-S-EAEFGGSG--          207
Bv3GGT-like2                     LPQPRKLAI-NCFSEED--MKQPPPGFPLP--SIRLRAHEARSLSSA-S-KAEFGGGG--          201
CqUGT79B30-like1                 CPFKRKE--SNNPTAAD--LVAPPPGFPSQ--SIWLRAQEAADVEAAPV-NTVYGPEH--          200
AhUGT79B30-like4                 APYKK-E--GYLPTAAD--LLRPPPGYPS---PIRMKPYEAKI-MAGAG-KTAFGLGG--          198
CqAmaSy2                         APYKKIP--GYHPNAND--LLTPPPEFPSQ--SIRMLPQEAEI-MAGAG-KTPFGLGG--          199
CqAmaSy1                         APYKKIP--GYHPNADD--LLTPPPEFPSQ--SIRMLPQEAEI-IAGAL-KTPFGLGG--          199
Bv3GGT-like1                     ISRITG---------------AYPPDLSSP--IFQMRAHEMKV-MEHIS-EKPFAPGG--          188
AhUGT79B30-like3                 AAYITA------PDHTN--TLDPPPGLSSP--IFKMQAHESRI-LSAVS-KRPFGSTG--          195
AhUGT79B30-like2                 AAYITA------PDHTD--ILGPPPGLSSP--IFKTRAHEARVGLSAVI-KKPFGLTG--          195
                                                                  .                   :

6BK0:A|PDBID|CHAIN|SEQUENCE      ---SIEQFAGLEDDDDVLVNSFSDLEPKEAAYMESTWRAKT---IGPSLPSFYLDDGRLR          257
CqUGT79B2-like                   VTFYERITTSLKSSNVIAIKTCREIEGKYCDYLSKQYDDKPILLSGLDLPEPQ-------          249
CqUGT79B6-like2                  VTFYERITTSLRCSDAIAIKTCREIEGKYCDFLSKQFN-KPILLSGLDLPKPNGP-----          273
AtF3G2GT                         TSFYERIMIGLKNCDVISIRTCQEMEGKFCDFIENQFQ-RKVLLTGPMLPEPD-------          238
AhUGT79B6-like                   MKFYDRVMAALTDCDVIAIRTCREIEADFCDYISNQYN-KPVFLTGPILSEPEP------          249
BvUGT97B6-like1                  LTFYDRMSTALKSCDAIAIRTCREIEDNFCNYISSQYN-KPVLLTGPVLPETN-------          248
BvUGT97B6-like2                  LTFYDRIVTAMKSCDAIAIRTCREIEDNFCDYISAQYN-KPVLLTGPVLPETN-------          248
CqUGT79B6-like1                  FTFYERITTALKSCDAIAIRTCREIEGNFCDYVSAQYN-KPVILTGPVLPENDN------          249
CqUGT79B6-like3                  LTFYERITTALKSCDAIAIRTCREIEGNFCDYVSAQYN-KPVILTGPVLPENDN------          249
IpA3G2Gt                         ITFFDRIFTAVSESDGLAYSTCREIEGQFCDYIETQFQ-KPVLLAGPALPVPS-------          249
GmF3G2Gt                         VLFYDRQFIALNEADLLAYRTCREIEGPYMDYIGKQFN-KPVVATGPVILDPP-------          247
CqUGT79B30-like5                 KTMLQRHAVALEACDAFAVKSCREMEGVYCDFIHKVYG-KPMLLAGPVVPEQP-------          257
BvUGT79B30-like                  KMMLQRHAVALEACDAFAAKSCREMEGDYCDFVYNVYR-KPLLLAGPVVPEPP-------          250
Cq3GGT-like2                     MTLVERKTKCFEECDLIASKTCREIEGAYYDFYENKLI-KPFLLVGPMMPEPP-------          254
CqUGT79B30-like2                 VPLLQKNATSFRECDVVGSKTSWEMEGPYCDFIKKQIN-KPVLLTGPMVPEPP-------          252
AhUGT79B30-like1                 LTLLERVEIAFKECDAIGVKTCREMEGTYCDYVQKNIK-KPVLLAGPVVPQMP-------          253
Cq3GGT-like1                     LSLLERVSIAFQQCDAVCIKTCREMEGPYCDFVQKNYR-KPVLLAGPVVPELP-------          259
Cq3GGT-like3                     LSLLERVSVAFQQCDAVCIKTCREMEGPYCDFVQKNYR-KPVLLAGPVVPELP-------          259
Bv3GGT-like2                     MSLLERVSEAFQQCDAVCIKTCREMEGSYCDFMQKNYG-KPVLLAGPVVPELP-------          253
CqUGT79B30-like1                 LTLLDRVNKSLRECDAIGAKSFKELEGAYCEYVEKVYG-KPVLLAGPLITKLA-------          252
AhUGT79B30-like4                 FTLAERLAVSFIECDAFGVKTCKEMEGEHCKFFEDVFG-KPVLLAGPVVPKLP-------          250
CqAmaSy2                         LTLAERLGVSFRECDAFGVKTCTEMEGEYCKFFEKIFG-KPVLLAGPMVPKTP-------          251
CqAmaSy1                         LTLAERLGVSFRECDAFGVKTCAEMEGEYCKFFEKIFG-KPVLLAGPMVPKRP-------          251
Bv3GGT-like1                     VTLPEMFRISFEECDAVGVKTCKEMEGIYCEFAEKTLD-KPVLLAGPVVPKLP-------          240
AhUGT79B30-like3                 KTMTEMFGISFRECDAIGAKTCTEMEGEYYEFVKKTLG-KPLLLAGPVVPIQP-------          247
AhUGT79B30-like2                 KTLIEMFGISFRECDAIGANTCMEMEGEYYEFVNKTLG-KPLLLAGPVVPIQP-------          247
                                           .   : .   :  ::*     :       :     *  :

6BK0:A|PDBID|CHAIN|SEQUENCE      SNTAYGFNLFRSTVPCMEWLDKQPPRSVVLVSYGTVSTFDVAKLEELGNGLCNSGKPFLW          317
CqUGT79B2-like                   --------EGELETRWENWLSQFKPGSVVICVFGSQVVLDVAQFQELLLVFEMTKLPFLI          301
CqUGT79B6-like2                  -------SETILEAHWTDWLSQFSPRSVVFCVFGSQVILDVAQFQELLLGFEMTNLPFLV          326
AtF3G2GT                         -------NSKPLEDQWRQWLSKFDPGSVIYCALGSQIILEKDQFQELCLGMELTGLPFLV          291
AhUGT79B6-like                   -------NRNSLNPNLADWLHTFDDKSVIFCAFGSQFIFEKDQFQELVLGFELTKKPFLV          302
BvUGT97B6-like1                  -------NNNPLEARWAEWLSRFEPNSVVFCAFGSQFILEKDQFQELLLGFEMTMVPFLV          301
BvUGT97B6-like2                  -------NNHPLEAQWVEWLSRFEPNSVVFCAFGSQYILEKDQFQELLLGFEMTMLPFLV          301
CqUGT79B6-like1                  -------SNNPLEAQWADWLCGFGPGSVVFCAFGSQFILERDQFQELLLGFEMTKLPFLV          302
CqUGT79B6-like3                  -------SNSPLEAQWADWLGGFGTGSVVFCAFGSQFILERDQFQELLLGFEMTKLPFLV          302
IpA3G2Gt                         --------KSTMEQKWSDWLGKFKEGSVIYCAFGSECTLRKDKFQELLWGLELTGMPFFA          301
GmF3G2Gt                         --------TLDLEEKFSTWLGGFEPGSVVYCCFGSECTLRPNQFLELVLGLELTGMPFLA          299
CqUGT79B30-like5                 --------AAVLDDHLDGWLSGFDKGSVIYCALGSECSLSKDRFQELLLGFELTGMPFYA          309
BvUGT79B30-like                  --------AARLDDKLNAWLSSFAKDSVIYCALGSECFLSKDRFQELLLGLELTGMPFLA          302
Cq3GGT-like2                     --------TSRLDDYFDGWLNGFGHGTVVYCALGSECVLGKDQFQELVYGLELTGRPFLA          306
CqUGT79B30-like2                 --------SSKLDDSLDCWLKGFGHGRVLYCALGSECVLKMDQFQELVLGLELTGRPFLA          304
AhUGT79B30-like1                 --------DDQLDEYFNTWLQGFGKDTVLYCALGSEASLGVLQFQELL------------          293
Cq3GGT-like1                     --------AGQLDEYFDNWLKDFGNGSVVYCALGSECFLQKDQFQELVLGLELTGKPFLA          311
Cq3GGT-like3                     --------AGQLDEYFDNWLKNFGNGSVVYCALGSECFLQKDQFQELVLGLELTGKPFLA          311
Bv3GGT-like2                     --------ATQLDEYFDRWLKGCGNGSVVYCALGSECTLQKDQFQELILGLELTGMPFLA          305
CqUGT79B30-like1                 --------SSKLDERIHGWLQGFDDASVIYCALGSECVMNLNHFQHLLLGLELTGRPFFV          304
AhUGT79B30-like4                 --------SSKLDEHFDEWLNGFDESSVIFCALGSECSLEINQFHELLLGLELTGRPFLA          302
CqAmaSy2                         --------SSKLDKYFDDWLNSFGNASVIYCALGSECALKLNQFQELVLGLELTGRPFLA          303
CqAmaSy1                         --------SSELDNYFDDWLNSFRTSSVIYCALGSECALNLNQFQELVLGLELTGRPFLA          303
Bv3GGT-like1                     --------SSKLDDYVDAWLSTFGSGTVIFCALGSECILEQNQFQDILLGLELTGKPFLA          292
AhUGT79B30-like3                 --------ASKLDEKVDEWLNGFGAETVIYCALGSECVLELSQFQQILLGLELTGRPFFA          299
AhUGT79B30-like2                 --------ASKLDEKVNEWLNGFGAETVVYCALGKS------------------------          275
                                                   **       *:    *.
```

```
6BK0:A|PDBID|CHAIN|SEQUENCE     VVRSNE-----EHKLSVQLRKKCEKRGLIV-PFCPQLEVLAHKATGCFLSHCGWNSTLEA      371
CqUGT79B2-like                  AFNPPMGCTTVEEAFPEGFVDRIGDRGVVTGEWVQQQQILAHTSVGCFVSHCGSGSIWES      361
CqUGT79B6-like2                 AFKPPTGYATVEEAFPEGFGRRVGDRGVVTGEWVQQPQILAHPSVGCFVSHCGSGSIWEC      386
AtF3G2GT                        AVKPPKGSSTIQEALPKGFEERVKARGVVWGGWVQQPLILAHPSIGCFVSHCGFGSMWEA      351
AhUGT79B6-like                  ALKPPTGCSTIEEALPNGFKERVRNRGKIVGDWVQQTLILGHPSIGCFVSHCGFGSMWEG      362
BvUGT97B6-like1                 ALKPPQGCGSVEEALPEGFKERVGERGVVHGGWVQQPLILAHPSVGCFVNHCGFGSMWES      361
BvUGT97B6-like2                 ALKPPQGCGFVEEALPEGFKERVGERGVVHGGWVQQPLILAHPSVGCFVNHCGFGSMWES      361
CqUGT79B6-like1                 ALKPPKGCGTIEEALPEGFKERVGDRGVVHGGWVQQPQILAHPSVGCFVNHCGFGSMWES      362
CqUGT79B6-like3                 ALKPPNGCATIEEALPEGFKERVGDRGVVHGGWVQQPQILEHPSVGCFVNHCGFGSMWES      362
IpA3G2Gt                        ALKPPFEAESIEAAIPEELKEKIQGRGIVHGEWVQQQLFLQHPSVGCFVSHCGWASLSEA      361
GmF3G2Gt                        AVKAPLGFETVESAMPEGFQERVKGRGFVYGGWVQQQLILAHPSVGCFITHCGSGSLSEA      359
CqUGT79B30-like5                ALKPPIGYETIESALPEGFIERTKGRGVVHGGWVQQQLILQHSSVGCFLTHCGAGSLSEA      369
BvUGT79B30-like                 ALKLPIGYETMEHALPEGFEERTKGRGIVHGDWVQQQLILQHPSVGCFVTHCGAGSLSEA      362
Cq3GGT-like2                    ALRPPSGCETIEQALPEGYMEKIKGRGIIHSGWVQQQLILQHPSVGCFVTHCGAGSLTEA      366
CqUGT79B30-like2                ALRPPSGCETIEQALPEGYMEKVKGRGIIHSGWVQQQLILHHPSVGCFVTHCGAGSLSEA      364
AhUGT79B30-like1                -------------ALPEGFAERTKGRGIIHIGWVQQHLILQHPSVGCFITHCGAGSLSEA      340
Cq3GGT-like1                    ALKLPTECSTLESALPEGFTERTKGRGIVHIDWVQQQLILQHPSVGCFLTHCGAGSLSEA      371
Cq3GGT-like3                    ALKLPTECSTLESALPEGFTERTKGRGIVHIGWVQQQLILQHPSVGCFITHCGAGSLSEA      371
Bv3GGT-like2                    ALKLPTECSTLESALPEGFKERTKGRGIVHIGWVQQQLVLQHSSVGCFLTHCGAGSLSEA      365
CqUGT79B30-like1                VLRPPTGYRTIESAFPKGIEERTKGRGMMHGGWVQQQLILQHPSVGCFVTHSGPGSISEA      364
AhUGT79B30-like4                ALKPPKNYKTIESALPEGFASRTRERGIVHEGWVQQQLILQHRSVGCFITHCGVGSLSEA      362
CqAmaSy2                        ALKPPMNYQTIESALPEGFAERTKSRGLIHGGWVQQQLIVQHPSVGCFITHCGAGSLSEA      363
CqAmaSy1                        ALKPPMNYQTIESALPEGLAERIKGRGLIHGGWVQQQLILQHPSVGCFITHCGAGSLSEA      363
Bv3GGT-like1                    ALKPPKNCKSLESGLPEGFSERVRGRGMIHGGWVQQQLILQHPSVGCFITHCGVGSLSEA      352
AhUGT79B30-like3                ALKPPKNYETIESALPKEFEERIKGKGIIDSGWVQQQLILKHPSIGCFITHCGVGSLSEA      359
AhUGT79B30-like2                -------------ALSEEFKERIKGKGIIDGGWVQQQLILKHPSVGCFVTHCGIGSLSEA      322
                                              :      :   :* :   :  *  .: * : ***:.*.*  *  *
```

```
6BK0:A|PDBID|CHAIN|SEQUENCE     IV-NGVPLVAMPHWQPTISKYVESLWGMGVRVQLDK-SGILQREEVERCIREVMDGDR 429
CqUGT79B2-like                  MM-SKNQIVLIPQLQDQVLFSKIFTTELKVAVEVDRGEDGYWVSRESLSKAIKSVMDDSS      420
CqUGT79B6-like2                 MM-SKNQIVLMPQLHEQFLFTKIFTTELKVAVEVERGEDGMWVSRESLSKAIKSVMGENS      445
AtF3G2GT                        LV-NDCQIVFIPHLGEQILNTRLMSEELKVSVEVKREETG-WFSKESLSGAVRSVMDRDS      409
AhUGT79B6-like                  LM-AKCQIVMVPQLGDQILNTRLMANEIEVGVEVERGENG-WVSKENLCKAIELVME-DN      419
BvUGT97B6-like1                 LM-AESQIVMVPQLGDQILNTRLMSGELVVGVEVERTENG-WVSKESLCKAIKSVMGEDS      419
BvUGT97B6-like2                 LM-AESQIVMVPQLGDQILNTRLMSGELVVGVEVERAESG-WVSKESLCKAIQSVMGEDS      419
CqUGT79B6-like1                 LM-AASQIVLVPQLGDQILNTRLMAGELKVAVEVEKAENG-WVSKESLCNAIKLVMDEKS      420
CqUGT79B6-like3                 LM-AASQIVLVPQLGDQILNTRLMAGELKVAVEVEKAENG-WVSKESLCNAIKLVMDEKS      420
IpA3G2Gt                        LV-NDCQIVLLPQVGDQIINARIMSVSLKVGVEVEKGEEDGVFSRESVCKAVKAVMDEKS      420
GmF3G2Gt                        LV-NKCQLVLLPNVGDQILNARMMGTNLEVGVEVEKGDEDGMYTKESVCKAVSIVMDCEN      418
CqUGT79B30-like5                MV-SDCQIVLCPQVTDQFINARMMSLDLKIGVEVEKGE-DGVLTKEGICKAIKAVMDLKS      427
BvUGT79B30-like                 MV-SNCQLVLFPQVTDQFINARFLSLDLKVGVEVEIGE-DGILTKDGICKTIKVIMDPKS      420
Cq3GGT-like2                    ML-SECQLVLIPQAIDQFVNARMMSLDLKVGVEVDKGEDDGFFTKQAVFEAIEMVMKPES      425
CqUGT79B30-like2                IL-SECQLVLIPQAVDQFVNARMMSLDLKVGVEVDKGEDDGFFTKQTVFEAIEMVMKPES      423
AhUGT79B30-like1                MV-TNCQLVMLPLVVDQSVNARMMSLDLKLGLEIQKRDDDGFFTKDALCKAVMIVMDTQN      399
Cq3GGT-like1                    IV-CDCQLVMLPHAVDQSINARLMSLDLKLGVEIEKRDEDGFFTREAVCKAVATVMEVEN      430
Cq3GGT-like3                    IV-CNCQLVMLPHAVDQSINARLMSLDLKLGVEIEKRDEDGFFTREAVCKAVATVMEVEN      430
Bv3GGT-like2                    MV-SQCQIIMLPQAVDQSVNARLMSLDLRLGVEVEMRDRDGFFTREAVCKAVVTVMDAEN      424
CqUGT79B30-like1                MISSECQLVLLPQILDQYLIARMMSLELKVGIEVEEC------TKEAISKAISTVMDGNN      418
AhUGT79B30-like4                MI-SKCQVVMIPQAIDQFINARMMSLEWKIGVEIETREDDGWFTREDVHKAITMVMDGES      421
CqAmaSy2                        MV-SECQVVLMPQAIDQFISARMMSLEWKVGVEVEKRKNDGLFTKEAVHKAVSLVMEEDS      422
CqAmaSy1                        MV-SECQVVLMPQAIDQFISARMMSLEWKVGVEVEKRKNYGLFTKEAVHKAVSLVMEEDS      422
Bv3GGT-like1                    MV-SQCQVVLMPQAVDQFMNARQMSLELKIGVEVESTETDGFFTREALCKAVSLVMDEQS      411
AhUGT79B30-like3                MV-SHCQVVFMPQAVDQFINARQMSLELKIGVEVESREEDGFYTKEAVSKAVSLVMDEHS      418
AhUGT79B30-like2                MV-SHCQMVFMPQAVDQFINARQMSLELKIGAEVESREEDGFYTKEAISKAVSSVMDEHS      381
                                ::     ::  *   :*   :: .     :. .:.         :: :   :  :*
```

```
6BK0:A|PDBID|CHAIN|SEQUENCE     KEDYRRNATRLMKKAKESMQEGG--SSDKNIAEFAAKYSN--------         467
CqUGT79B2-like                  EICGLVKKNH--EIWRQKLAQPG--LMSGYFDKFVKDLQALVSNNNVI         464
CqUGT79B6-like2                 EISGLVKKNH--EIWRQKLAQPV--LMSGYFDKFVKDLQVLVSNNVV-         488
AtF3G2GT                        ELGNWARRNH--VKWKESLLRHG--LMSGYLNKFVEALEKLVQNINLE         453
AhUGT79B6-like                  EVSFRVRKNH--AKWRDVMISER--FMKDYVDNFVKDLENLVG-----         458
BvUGT97B6-like1                 EVGCLVRNNH--ANWRQIMVSQG--FMRGYIDSFVKDLELLMG-----         458
BvUGT97B6-like2                 EVGCLVRNNH--AKWRQIMVSQG--FMRGYIDNFVKDLELLMG-----         458
CqUGT79B6-like1                 EVGCVIKNNH--AKWRQIMASQG--FMKGYIDNFIMDLEKIMN-----         459
CqUGT79B6-like3                 EVGCLIKNNH--VKWRQIMISHG--FMKGYIDNFIMDLEKIMT-----         459
IpA3G2Gt                        EIGREVRGNH--DKLRGFLLNAD--LDSKYMDSFNQKLQDLLG-----         459
GmF3G2Gt                        ETSKRVRANH--ARIRELLLNKD--LESSYVDSFCMRLQEIVEGI---         459
CqUGT79B30-like5                EVGREARINQ--AKWREFLLAKE--LEEFYISGFVENLLDLVT-----         466
BvUGT79B30-like                 EVGREARINR--VKWRDFLLAKG--LEASYISGFIQNLRGLVG-----         459
Cq3GGT-like2                    VVGKEVRDNH--TKLRNLLSKEG--LEDSYIDKFVQSLQDLV------         463
CqUGT79B30-like2                VVGKEVRDNH--TKLRNLLSKEG--LEDSYIDKFVQSLQDLV------         461
AhUGT79B30-like1                IMAKQLKTNH--TKWKEFLLKQG--VEDAYITAFIHSLQRLLTSS---         440
Cq3GGT-like1                    EVGREVRANH--AKWREFLLRDG--LEEAYISGFIQSLKGILV-----         469
Cq3GGT-like3                    EVGREVRANH--AKWREILLRDG--LEEAYISGFIQSLKDILV-----         469
Bv3GGT-like2                    EVGREVRANH--AKWREFLLKEG--LEDAYINGFIQSLQDLLSTQV--         466
CqUGT79B30-like1                DVAKEVRANR--VKWRKYLLREGPRLEESYISSFIYNLQKLLE-----         459
AhUGT79B30-like4                DVGREVRANH--AKWRDFILTQG--VEDSYISSFIESLQQLLIV----         461
CqAmaSy2                        EVGRDVRANH--AKWREFILTEG--LEDSYISSFIMSLQQLIGS----         462
CqAmaSy1                        EVGRDVRANH--AKWREFILTEG--LEDSYISSFIQSLQQLIGS----         462
Bv3GGT-like1                    EVAREVKANH--AKWRDFILTEG--LEDSYISSFIQSLQHLLQMN---         452
AhUGT79B30-like3                EVGREVRAKH--AKWRDFILKQG--LEDSYITSFINSLRQLL------         456
AhUGT79B30-like2                EVGREVRANH--AKWRDFILKQG--LEDSYIISFINSLQQLLH-----         420
```

Figure S6. Comparison of the deduced amino acid sequences of flavonoid 2”Gt 2”Rt 6”Rt cluster and the unknown cluster (Figure 2). Red characters indicate amino acid residues expected to be involved in activity. The gray background indicates the amaranthin synthetase group.

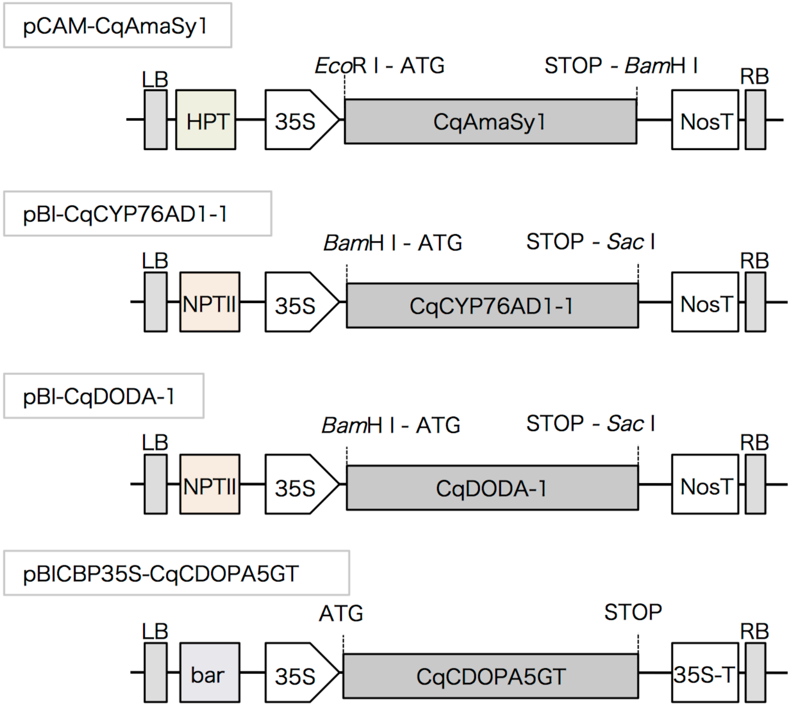

Figure S7 Schematic representations of the plant expression vectors. CqAmaSy1, *CqAmaSy1* CDS; CqCYP76AD1-1, *CqCYP76AD1-1* CDS; CqDODA-1, *CqDODA-1* CDS; CqCDOPA5GT, *CqCDOPA5GT* CDS; 35S, CaMV 35S promoter; NosT, *nopaline synthase* terminator; 35S-T, 35S terminator; RB, right border; LB, left border; HPT, *hygromycin phosphotransferase* expression cassette; NPTII, *neomycin phosphotransferase II* expression cassette; bar, *bar* expression cassette; ATG, start codon; and STOP, stop codon.

(a)

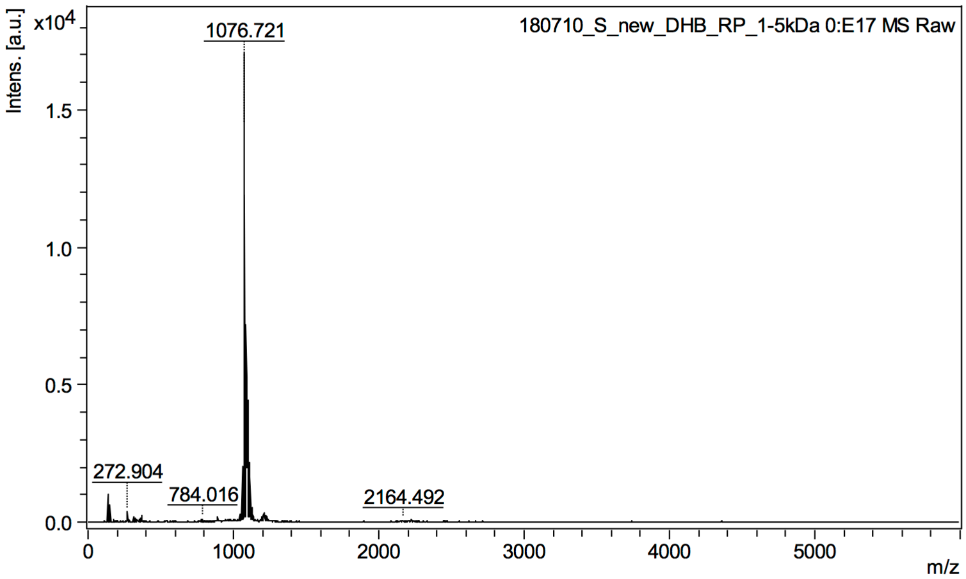

(b)

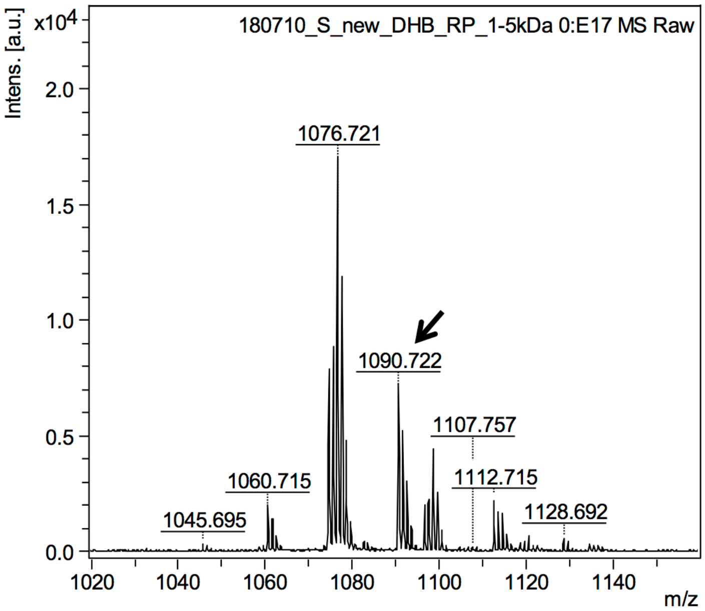

Figure S8 Characterization of HIV-1 protease substrate. (a) MS spectrum of HIV-1 protease substrate (Lys-Ala-Arg-Val-Nle-p-nitro-Phe-Glu-Ala-Nle amide, red arrows in Figure 7a). (b) Enlarged spectrum of (a). Arrow indicates HIV-1 protease substrate (*Mr* = 1090.637). The horizontal axis indicates the mass-to-charge ratio (*m/z*) and the vertical axis indicates the relative abundance.
