# Supplementary material for: Isolation of amaranthin synthetase from Chenopodium quinoa and construction of an amaranthin production system using suspension‐cultured tobacco BY‐2 cells
Source: Plant Biotechnol J. 2018 Dec 5;17(5):969–81. doi: 10.1111/pbi.13032 (PMC6587806; doi:10.1111/pbi.13032)
Supplement: Supplementary file 2 — Table S1 Proteins used for phylogenetic analysis. Table S2 Primers used in this study. [file PBI-17-969-s002.pdf]

Table S1. Proteins used for phylogenetic analysis

NCBI Accession No.	Gene Name	Species
CAA50376	Ph3RT	*Petunia hybrida*
ABA18631	Cs1,6RhaT	*Citrus sinensis*
BAN91401	GmF3G6“Rt	*Glycine max*
BAD95881	IpA3G2”GT	*Ipomoea purpurea*
BAR88077	GmF3G2“Gt	*Glycine max*
NP_200212	AtF3G2”GT	*Arabidopsis thaliana*
BAD77944	BpUGAT	*Bellis perennis*
BAH80312	CaUGT3	*Catharanthus roseus*
AAL06646	Cm1,2RhaT	*Citrus maxima*
BAU68118	ABRT2	*Lobelia erinus*
BAU68119	ABRT4	*Lobelia erinus*
AAM13132	AtUGT89C1	*Arabidopsis thaliana*
AEC09298	AtUGT73C6	*Arabidopsis thaliana*
BAA83484	SbUBGT	*Scutellaria baicalensis*
XP_021719267	CqUGT79B6-like1	*Chenopodium quinoa*
XP_021726554	Cq3GGT-like1	*Chenopodium quinoa*
XP_021731181	CqUGT79B2-like	*Chenopodium quinoa*
XP_021735671	CqUGT79B30-like1	*Chenopodium quinoa*
XP_021735839	Cq3GGT-like2	*Chenopodium quinoa*
XP_021735840	CqUGT79B30-like2	*Chenopodium quinoa*
XP_021735841	CqAmaSy2	*Chenopodium quinoa*
XP_021747968	CqUGT79B6-like2	*Chenopodium quinoa*
XP_021754077	CqAmaSy1	*Chenopodium quinoa*
XP_021758620	CqUGT79B30-like5	*Chenopodium quinoa*
XP_021766006	Cq3GGT-like3	*Chenopodium quinoa*
XP_021773738	CqUGT79B6-like3	*Chenopodium quinoa*
XP_010695817	Bv3GGT-like1	*Beta vulgaris*
XP_010686377	Bv3GGT-like2	*Beta vulgaris*
XP_010666234	BvUGT97B30-like	*Beta vulgaris*
XP_010674067	BvUGT97B6-like1	*Beta vulgaris*
XP_010675464	BvUGT97B6-like2	*Beta vulgaris*
Phytozome Accession No.	Gene Name	Species
AH008037-RA	AhUGT79B6-like	*Amaranthus hypochondriacus*
AH008346-RA	AhUGT79B30-like1	*Amaranthus hypochondriacus*
AH018627-RA	AhUGT79B30-like2	*Amaranthus hypochondriacus*
AH018628-RA	AhUGT79B30-like3	*Amaranthus hypochondriacus*
AH018629-RA	AhUGT79B30-like4	*Amaranthus hypochondriacus*

Table S2. Primers used in this study

			Sequence (5′→ 3′)
RT-PCR			
Quinoa hypocotyl expression			
	Cq3GGT-like1	Forward	ATGTCCAAGGAAAATGGCATTGCCAATGGC
		Reverse	TCATACGAGAATACCTTTCAGACTCTGTAT
	Cq3GGT-like3	Forward	ATGTCCAAGGAAAATGGCATTGCTAATGGCAAT
		Reverse	TTATACGAGAATGTCTTTCAGACTCTGTATGAACC
	Cq3GGT-like2	Forward	ATGTCATCATCAAACAATAACAATGGCAAGACTT
		Reverse	TCAAACCAAATCTTGTAGACTTTGAACAAACTT
	CqUGT79B30-like2	Forward	ATGTCAAAGATTAACGAAACCAATGAATGT
		Reverse	TCAAACCAAATCTTGTAGACTTTGAACAAACTT
	CqAmaSy2	Forward	ATGTCACAAAACAAAGACACCCAAATTCTA
		Reverse	TCATGATCCAATCAATTGTTGCAAACTCATA
	CqAmaSy1	Forward	ATGTCACAAAACAAAGACAACCAAA
		Reverse	TTATGATCCTATCAATTGTTGCAAACTCTG
	CqUGT79B30-like1	Forward	ATGTCTAACAACAAAAACTCCAAAATTCTAAAAG
		Reverse	TCACTCAAGCAACTTTTGTAGATTATAAATGAAGC
	CqUGT79B30-like5	Forward	ATGGATAAAAAAATAGCAAGTATGGTTGAGGAAAAAG
		Reverse	TCATGTAACTAGATCTAGTAGATTTTCAACA
	CqCYP76AD1-1	Forward	ATGGATCATGCAACACTAGCAATGAT
		Reverse	TCAATACCTAAGAACGGGAATAATCT
Infiltrated *N. benthaminana* leaves and transgenic tobacco BY-2 cell lines			
	Cq3GGT-like2	Forward	ATGTCATCATCAAACAATAACAATGGCAAGACTT
		Reverse	TAGGTGGTCGTAATGCTGCTAAAAATGGCC
	CqAmaSy1	Forward	CCAATGGTACCTAAACGGCCATCTTCGGAAC
		Reverse	TTATGATCCTATCAATTGTTGCAAACTCTG
	CqUGT79B30-like1	Forward	ATGTCTAACAACAAAAACTCCAAAATTCTAAAAG
		Reverse	GTGATTAAAGGCCCTGCTAAAAGCACAGGC
	CqAmaSy2	Forward	ACTTGTTGGATGCCCGAACTAGCCCGAAA
		Reverse	TCATGATCCAATCAATTGTTGCAAACTCATAAT
	CqCYP76AD1-1	Forward	AAAAGGTCACCTCAATACCTAAGAACGGGAATAATCT
		Reverse	TCAATACCTAAGAACGGGAATAATCTG
	CqCDOPA5GT	Forward	GGAGCAATCTACCTCACCGGAATGTCGGC
		Reverse	CCACCAGTAGCCTCATGCCTCAAAATATCCAACT
	CqDODA1-1	Forward	TGATACTATCTACGATTTTGATGACT
		Reverse	TTCATATGTATTCACTTCTTCAAACC
	AhUGT79B30-like3	Forward	ATGGGTTACAAAGAAGAGTCCTCTG
		Reverse	TTAAAGCAATTGTCGTAAACTATTG
	AhUGT79B30-like4	Forward	ATGTCTCACAACAAAGAATCCAACCC
		Reverse	CTATACAATTAACAATTGTTGTAAAC
	CbBetanidin-5GT	Forward	ATGGGTACACACTCAACAGCACCAGATC
		Reverse	CTACTCTTTCTCTGAAGTATGATAACCCT
	AcGFP1	Forward	GGATCCATGGTAAGCAAAGGAGCCGAACTTT
		Reverse	GGTCACCTCACTTATACAGCTCATCCATCCC
	L23	Forward	AGCTGATCCGTCCAAAAAATCTGATCCCA
		Reverse	TTTGTTGGCCACGTCCAACGCATCGTAG
	CesA	Forward	GAAGGTTGGACT ATGCAAGA
		Reverse	ATAGATCCATCCAA TCTCTTTTCCCCA
Direct sequence			
Check for the expressed gene of *CqAmaSy1*			
	CqAmaSy1	Forward	CCAATGGTACCTAAACGGCCATCTTCGGAAC
		Reverse	GGTCTATYTGGAAAGCTTAAATAGATG
		Seqence	CCATAAGGCGGTCTCCTTGGTGATGGAA
Vector construction			
Over-expressiong vector			
	CqAmaSy1	Forward	AAAGAATTCATGTCACAAAACAAAGACAACCAAA
		Reverse	AAAGGATCCTTATGATCCTATCAATTGTTGCAAACTCTG
	CqAmaSy2	Forward	AAAGAATTCATGTCACAAAACAAAGACACCCAAATTCTA
		Reverse	AAAGGATCCTCATGATCCAATCAATTGTTGCAAACTCATAAT
	CqUGT79B30-like1	Forward	AAAAAGCTTATGTCTAACAACAAAAACTCCAAAATTCTAAAAG
		Reverse	AAAGGATCCTCACTCAAGCAACTTTTGTAGATTATAAATGAAGC
	Cq3GGT-like2	Forward	AAAGAATTCATGTCATCATCAAACAATAACAATGGCAAGACTT
		Reverse	AAAGGATCCTCAAACCAAATCTTGTAGACTTTGAACAAACTT
	CqCYP76AD1-1	Forward	AAAGGATCCATGGATCATGCAACACTAGCAATGAT
		Reverse	AAAGAGCTCTCAATACCTAAGAACGGGAATAATCT
	CqDODA-1	Forward	AAAGGATCCATGAAAATGGTTGTTGAAGAGGGAAAAAATAACGAAATG
		Reverse	AAAGAGCTCTTAACTGGAAGTGAACTTGTAAGCGCCATGACATAGG
	CqCDOPA5GT	Forward	ATGGAGTCATCAACAAAAGAACAAG
		Reverse	TCAAGCATCTTCTTTAGATTTCTTTCCCCT
	Bv3GGT-like1	Forward	AAAGAATTCATGGGTGAAAACAAAGAATCGCAAG
		Reverse	AAAGGATCCTCAGTTCATCTGAAGCAAGTGTTGTAGAC
	AhUGT79B30-like3	Forward	AAAGAATTCATGGGTTACAAAGAAGAGTCCTCTG
		Reverse	AAAGGATCCTTAAAGCAATTGTCGTAAACTATTG
	AhUGT79B30-like4	Forward	AAAGAATTCATGTCTCACAACAAAGAATCCAACCC
		Reverse	AAAGGATCCCTATACAATTAACAATTGTTGTAAAC
	DbB5GT	Forward	AAAGGATCCATGGGTACACACTCAACAGCACCAGATC
		Reverse	AAAGGTCACCCTACTCTTTCTCTGAAGTATGATAACCCT
